# Improved spatial resolution by induced live cell and organelle swelling in hypotonic solutions

**DOI:** 10.1038/s41598-019-49408-2

**Published:** 2019-09-09

**Authors:** Astha Jaiswal, Christian H. Hoerth, Ana M. Zúñiga Pereira, Holger Lorenz

**Affiliations:** 1 Center of Molecular Biology, University of Heidelberg (ZMBH), Heidelberg, Germany; 2 Present Address: Instituto Clodomiro Picado, Facultad de Microbiología, Universidad de Costa Rica, San José, Costa Rica

**Keywords:** Fluorescence imaging, Super-resolution microscopy

## Abstract

Induced morphology changes of cells and organelles are by far the easiest way to determine precise protein sub-locations and organelle quantities in light microscopy. By using hypotonic solutions to swell mammalian cell organelles we demonstrate that precise membrane, lumen or matrix protein locations within the endoplasmic reticulum, Golgi and mitochondria can reliably be established. We also show the benefit of this approach for organelle quantifications, especially for clumped or intertwined organelles like peroxisomes and mitochondria. Since cell and organelle swelling is reversible, it can be applied to live cells for successive high-resolution analyses. Our approach outperforms many existing imaging modalities with respect to resolution, ease-of-use and cost-effectiveness without excluding any co-utilization with existing optical (super)resolution techniques.

## Introduction

**Figure 1:**
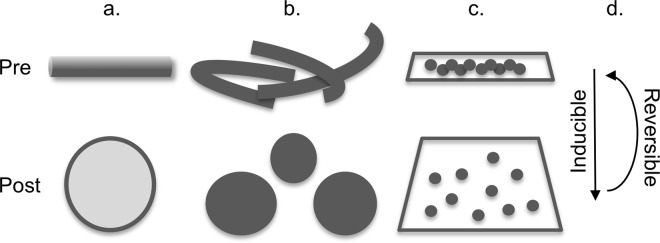
Overview of the key benefits of induced morphology changes of cells and organelles. Graphical illustrations are shown pre (top row) and post (bottom row) swelling induction. (**a**) Non-isotropic swelling enlarges otherwise non-resolvable biological structures for improved resolution, for example, of membrane (dark gray) vs. lumen (light gray) protein locations, as shown in Figs 4, 5. (**b**) Non-isotropic swelling leads to morphological changes for improved separation and quantification of objects, as shown in Fig. 7b,c. (**c**) Swelling enlarges the volume of cells for improved separation and quantification of clumped intracellular objects, as shown in Fig. 7a. (**d**) Swelling of cells is inducible and reversible for transient analyses, as shown in Fig. 6 and Fig. 7c.

Scientific bioimaging aims to reveal scientifically relevant information. Besides the mere metrics of biomaterial like cells, organelles, or even sub-organelle structures, more informative are data addressing protein sub-locations and interactions. Especially in the spatio-temporal context of cellular dynamics the precise protein sub-location within the membranous organelle system of the cell is crucial. To know that a certain protein is located within a particular organelle is often not sufficient. More precise information is needed, for example whether the protein is membrane-bound, or not. This puts a technical challenge on bioimaging since many organelles’ membrane assemblies are well below the micrometer range in size and distance. Especially superresolution light microscopy is able to provide such data from fixed samples and, at often lower spatial resolution, from live cells^1–6^. In any case, whether a certain biological structure is resolvable or not depends on two separable factors: the resolution limit of the chosen optical instrument and the size of the biological structure. If biostructures are too small for a given optical instrument to resolve, scientists can either try to find answers by using related technologies (e.g. electron microscopy, Förster resonance energy transfer etc.) or have to wait until new and often expensive technological advances provide solutions. Recently, a new approach for improving spatial resolution has been introduced which puts the focus away from the instrument onto the sample itself. Expansion microscopy^7^ argues that by isotropically expanding biomaterial, otherwise hidden structural information could be lifted into the resolvable range. Numerous studies have since shown that expansion microscopy can indeed be successfully applied to cells and tissues^8^. We asked if this idea of modifying the sample rather than the optical instrument could be explored further and extended to live cells. Since expansion microscopy relies on progressive enlargements of fixed biomaterial, its protocol is rather time-consuming (up to several days) and obviously unsuitable for live cells^9^. However, live cells have the ability to regulate their volume, which has been extensively reviewed^10^. Moreover, both live mammalian cells and many of their organelles can easily be enlarged by exposing them to hypotonic solutions^11^. In short, hypotonic solutions cause water influx into the cell’s interior to equal osmotic concentrations. The resulting swelling of the cell and organelles is not isotropic, which can be very beneficial from an imaging point-of-view (Fig. 1 and Supplementary Table 1). Although swelling can be induced similarly in yeast^12^ and bacteria spheroplasts^13^, we here focus on the many benefits of induced mammalian cell and organelle swelling in hypotonic solutions. We show that this approach provides a fast and reliable way to determine exact protein sub-locations and organelle quantities that are otherwise very difficult to achieve. As proof of concept we demonstrate precise determinations of protein locations on endoplasmic reticulum (ER) membranes and lumens, and mitochondria inner/outer membranes and matrixes, all performed within a few minutes on a conventional widefield or confocal microscope at no extra costs. We also demonstrate that cell swelling is suitable for live cells. Mammalian cells recover from the swelling when replaced in regular culture media, allowing for multiple transient analyses of the same cell(s) over time.

## Results

### Expansion of live cells and organelles in hypotonic solutions

**Figure 2:**
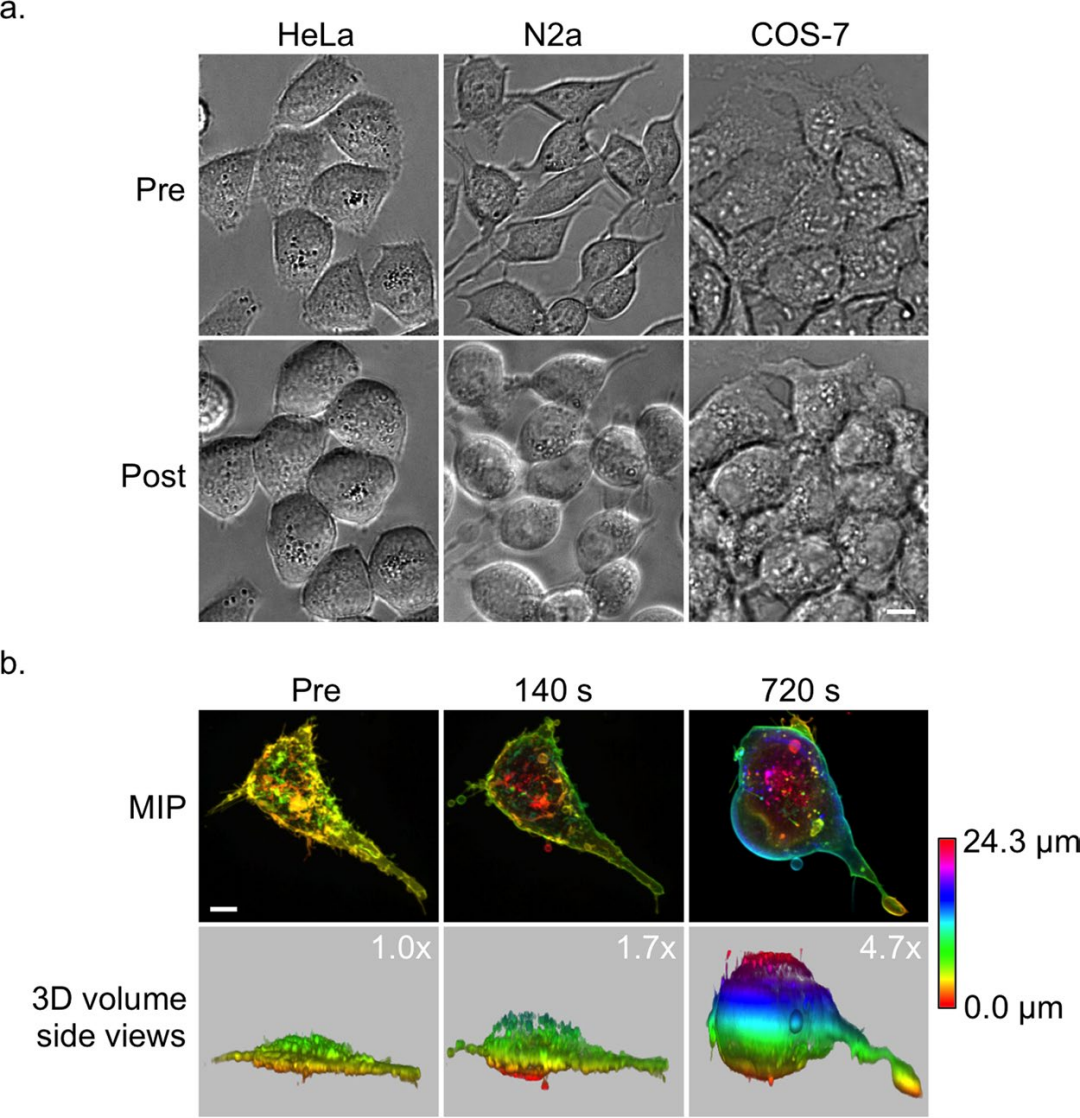
Swelling of live cells in hypotonic solutions. (**a**) Differential interference contrast (DIC) images of live HeLa (left), N2a (middle) and COS-7 (right) cells pre (top row) and post (bottom row) incubation in diH_2_O at time point 256 s. (**b**) MIP images (top row) and side views of 3D volume renderings (bottom row) of a COS-7 cell expressing MyrPalm-mEGFP pre (left) and post incubation in diH_2_O at time points 140 s (middle) and 720 s (right). The Z-layers of the 3D image data are color-coded. The volume increase of the cell is indicated in the top right corners (bottom row). Scale bars, 10 µm.

To test the effect of hypotonic solutions on mammalian cells we used six different cell lines (COS-7, HEK 293, HeLa, N2a, RPE-1, U-2 OS) and exposed them to deionized water (diH_2_O). We did this by simply replacing the culture media with diH_2_O whilst the cells were imaged by light microscopy. All cell lines reacted similar to diH_2_O and grew in size, especially in height (Fig. 2a and Supplementary Video 1). The swelling started almost instantaneously and continued until the cells reached a maximum size (Fig. 2b). From the six tested cell lines only one, N2a, showed a tendency to burst at the cell membrane by succumbing to osmotic pressure. We could easily fix this problem by using a 4:1 (v/v) diH_2_O/culture media mix instead of straight diH_2_O to keep N2a cells as intact as the other cell lines.

**Figure 3:**
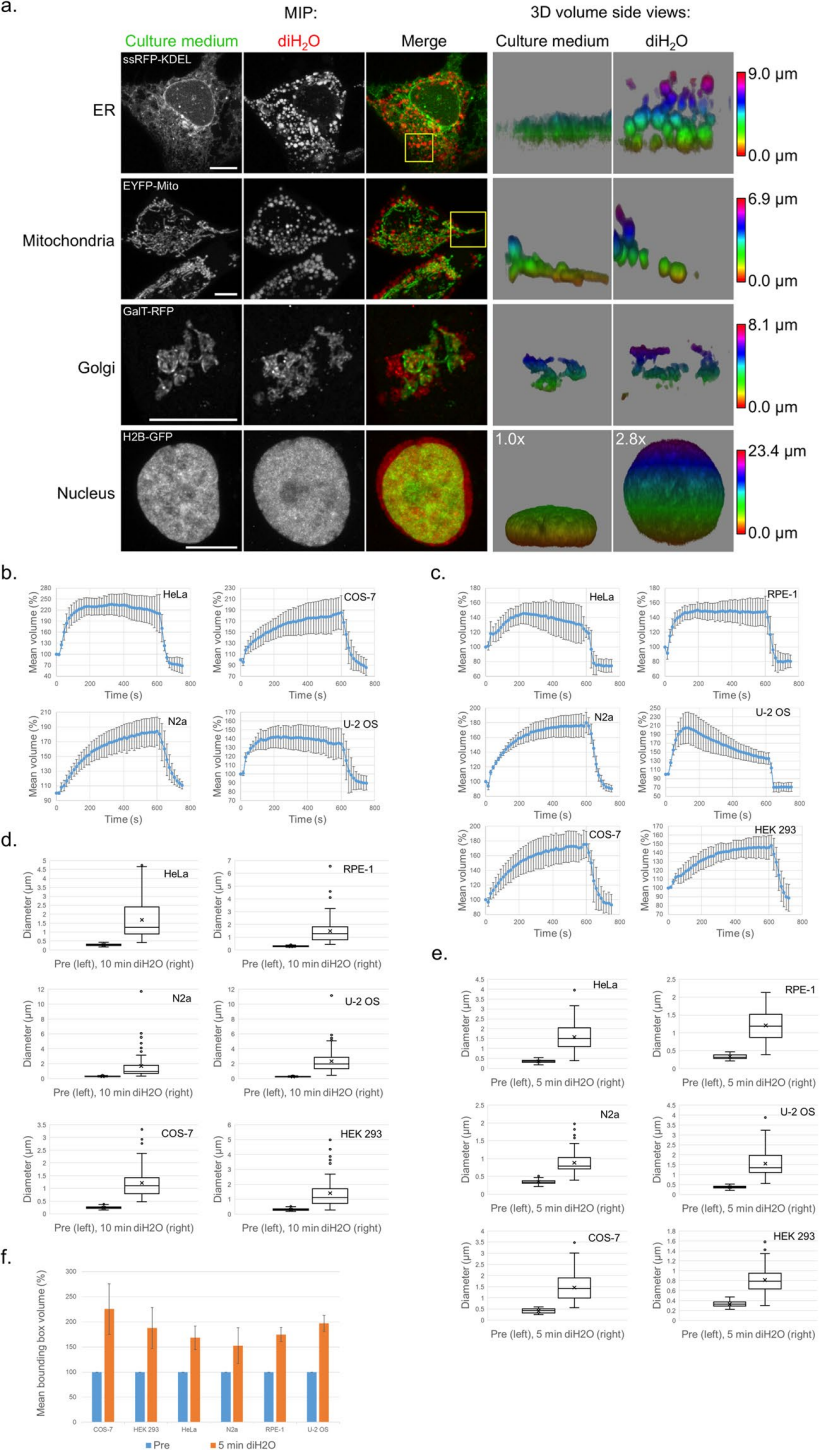
Swelling of live organelles in hypotonic solutions. (**a**) MIP images (columns 1–3) and corresponding side views of 3D volume renderings (columns 4 and 5) of a U2-OS cell expressing ssRFP-KDEL (top row), HeLa cells expressing EYFP-Mito (row 2), a HeLa cell expressing GalT-RFP (row 3) and a COS-7 cell expressing H2B-GFP (bottom row) pre (culture medium, columns 1 and 4) and post incubation in diH_2_O for 200 s (diH_2_O, columns 2 and 5). The Z-layers of the 3D volume renderings are color-coded. The volume increase of the nucleus is indicated in the top left corners (bottom row, columns 4 and 5). Scale bars, 10 µm. (**b**,**c**) Quantitative analyses of (**b**) whole cell volumes and (**C**) nucleus volumes over time upon incubation of cells in diH_2_O. The hypotonic solution was applied to the cells at 0 s and replaced with conventional cell culture media at 600 s to address swelling and unswelling dynamics. Shown are mean volumes of (**b**) cells and (**c**) nuclei ± standard deviations from independent experiments of individual cells, (**b**) HeLa, n = 8, N2a, n = 6, COS-7, n = 6, U-2 OS, n = 7, (**c**) HeLa, n = 7, N2a, n = 8, COS-7, n = 8, U-2 OS, n = 6, RPE-1, n = 7, HEK 293, n = 7. For both, cell and nucleus volume quantification, 3–4 replicates from independent experiments were used. (**d**–**f**) Quantitative analyses of (**d**) ER and (**e**) mitochondria diameters and (**f**) Golgi 3D shapes pre and post incubation of cells in diH_2_O. Post incubation image series were taken at 10 min (**d**) and 5 min (**e**,**f**). Shown are box plots from plot profile measurements of ER (**d**) and mitochondria (**c**) membranes, or 3D bounding box volume measurements of the Golgi ± standard deviations from independent experiments of individual cells. (**d**) n = 54–79, 3–4 replicates, (**e**) n = 46–73, 3 replicates (**f**) n = 3–5, 3 replicates.

Having established that mammalian cells can readily increase in size upon exposure to hypotonic solutions, we wanted to look closer at the consequences on intracellular organelle and membrane sizes. All cell lines were transiently transfected in order to express fluorescent protein (FP) fusions of marker proteins for the following cellular components: plasma membrane, ER, mitochondria, Golgi apparatus and the nucleus. To image volumes, Z-sections of the cells were recorded on a confocal microscope pre and post incubation in hypotonic solutions. For better appreciation of the gain in size and volume, the three-dimensional (3D) image data were presented either as color-coded maximum intensity projections (MIP) or as texture-based volume renderings. By doing so, considerable swellings of the plasma membrane, mitochondria, ER and nucleus could be visualized (Fig. 2b and Fig. 3a and Supplementary Fig. S1). Since the plasma membrane marker MyrPalm-mEGFP^14^ (for myristoylated and palmitoylated mEGFP) outlined the whole cell, the extent of swelling could be measured for individual cells. Even though swelling varied among cells within and between cell lines, all cells enlarged their volumes significantly. At about 4–12 min of incubation in hypotonic solutions the maximum swelling state was reached and the cells stopped expanding. The gain in volume ranged from approximately 1.5 to 5 times compared to the original volumes (Fig. 2b). Moreover, the swelling was not restricted to the outer membrane. The common worm- and netlike anatomies of mitochondria and the ER, respectively, changed significantly as shown by the ER luminal protein ssRFP-KDEL^15^ and EYFP-Mito, a fusion of EYFP and the targeting sequence from subunit VIII of human cytochrome c oxidase, which targets it to the mitochondria matrix^16^. Both organelles displayed rounded structures in diH_2_O that appeared more voluminous (i.e., blown) in comparison to their slimmer and more tubular native phenotypes (Fig. 3a, rows 1 and 2 and Supplementary Fig. S1). Larger volumes, even if slightly more moderate, could also be observed for the Golgi apparatus upon diH_2_O incubation. By using the Golgi transmembrane protein galactosyltransferase (GalT) tagged to RFP (GalT-RFP)^17^, an increase in size and spread of the Golgi constituents was detectable for all cell lines tested (Fig. 3a, row 3 and Supplementary Fig. S1). Also, cell nuclei, labeled with the histone 2B-FP marker protein H2B-GFP^18^, expanded up to 3 times in volume in hypotonic solutions compared to their normal size in cell culture media (Fig. 3a, bottom row and Supplementary Fig. S1). Since whole cells and nuclei have 3D volume sizes that are resolvable by diffraction-limited microscopy, we performed volume quantifications upon incubation in diH_2_O over time. We measured whole-cell volumes of HeLa, N2a, COS-7 and U-2 OS cells, which, unlike RPE-1 and HEK 293 cells, had cell borders regular enough to be segmented for volume quantifications. To perform the analysis, 3D image data (Z-stacks) of cells expressing the plasma membrane marker MyrPalm-mEGFP and the cytosol marker mCherry were acquired upon incubation in diH_2_O. Graphs of the mean cell volume over time clearly show that all cells enlarged their volumes, but the dynamics of swelling varied between the cell lines (Fig. 3b). HeLa and U-2 OS cells expanded more rapidly than N2a and COS-7 cells, which grew more steadily in volume over the diH_2_O incubation period of 10 min. Furthermore, a noticeable decrease in U-2 OS cell volume could be observed indicating volume compensatory actions of these cells against the osmotic pressure. This was also observable for U-2 OS nuclei, which showed a similar reduction in nuclear volumes (Fig. 3c) over time in the presence of diH_2_O. Again, the different cell lines displayed different swelling dynamics of the nuclear H2B-GFP signals, with N2a, COS-7 and HEK 293 cells showing more steadily growing volumes. Overall, the volume quantification results indicate that all cell lines (and their nuclei) reacted to diH_2_O by swelling. The dynamics and extent of swelling, however, not only varied to some degree within a particular cell line, but also showed some cell line-specific characteristics.

**Figure 4:**
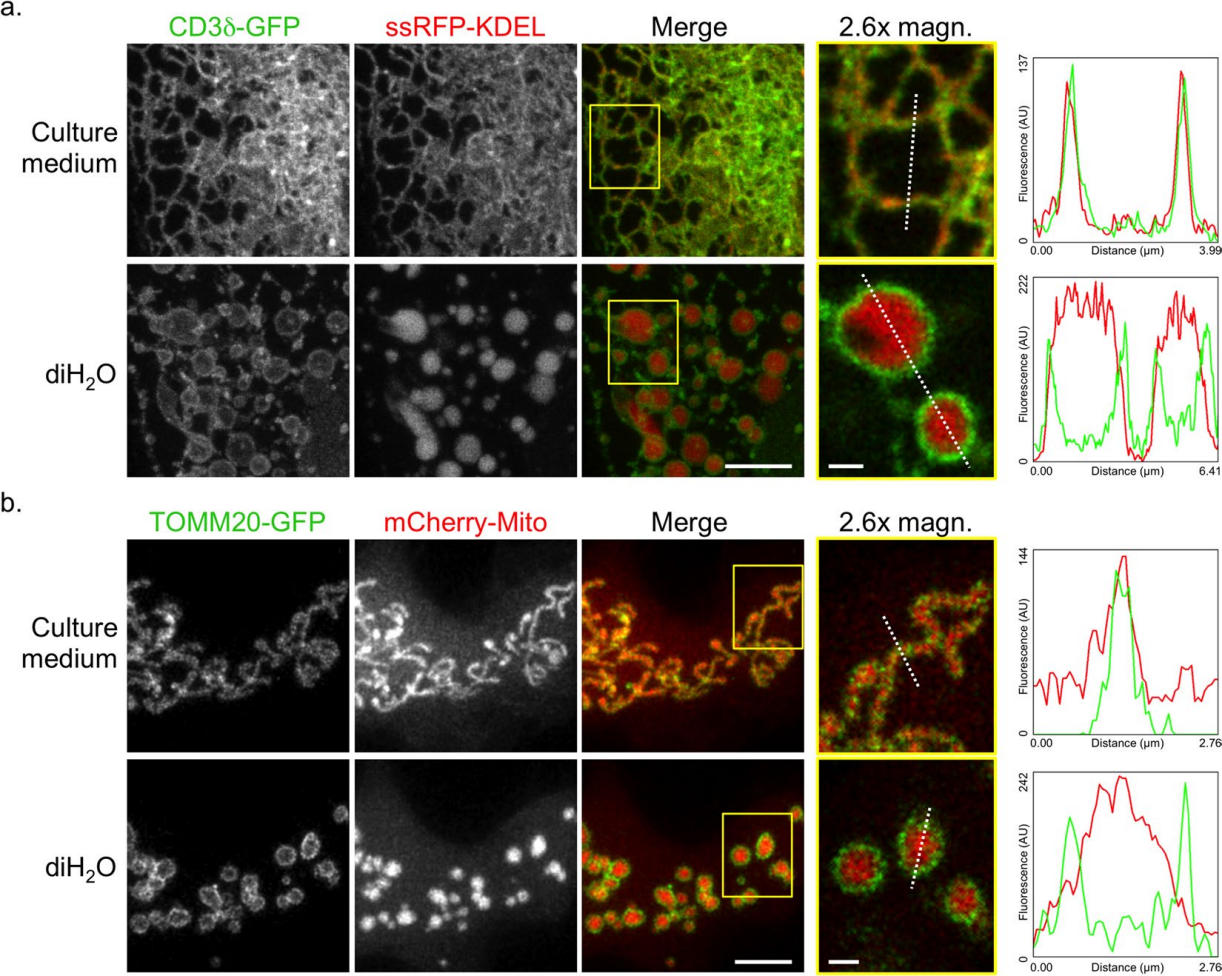
Improved protein location determination upon organelle swelling. (**a**,**b**) MIP images (columns 1–3) and single slice images (column 4) of (**a**) a U2-OS cell co-expressing CD3δ-GFP (green) and ssRFP-KDEL (red) and (**b**) a COS-7 cell co-expressing TOMM20-GFP (green) and mCherry-Mito (red). The single slice images (column 4) are 2.6x magnifications (magn.) of the corresponding area outlined in column 3 (merge, yellow rectangle). Profiles of fluorescence intensity (arbitrary units) taken along the white dotted lines (column 4) are presented (right). ER (**a**) and mitochondria (**b**) signals are shown pre (culture medium, top rows) and post incubation in diH_2_O for 200 s (diH_2_O, bottom rows). Scale bars, 5 µm (column 3), 1 µm (column 4).

Next, we quantitatively assessed the size increase of the ER, mitochondria and the Golgi of the six cell lines upon swelling. Unlike whole cells and nuclei, the volumes of ER tubular/sheet structures, small mitochondria and Golgi cisternae are below the resolvable range of diffraction-limited microscopy. Therefore we measured the increase of 2D diameter distances within the ER and mitochondria and the spread of the Golgi 3D shape, respectively, upon diH_2_O incubation (Fig. 3d–f). 3D image data (Z-stacks) of cells expressing the ER membrane marker CD3δ-GFP^19^, the outer mitochondrial membrane (OMM) protein TOMM20-GFP^20^ and the Golgi marker GalT-RFP were acquired pre and post incubation in diH_2_O. For the ER and mitochondria, plot profiles were taken from single slices of the Z-stacks to determine the diameter increase within the organelles upon swelling (exemplified in Fig. 4, see plot profiles’ green lines). For the Golgi, due to its rather inconsistent shape, the 3D GalT-RFP signal was segmented and the increase of the Golgi size and spread upon swelling was analyzed by the growth of its corresponding 3D bounding box^21^ (Fig. 3f and Supplementary Fig. S1e). Similar to the whole cell and nucleus measurements, the ER, mitochondria and Golgi showed a clear increase in size upon incubation in diH_2_O, and also some variability within and across cell lines. Especially for the ER and mitochondria, the results demonstrate a significant enlargement of the organelles from sub-micrometer diameters way into the micrometer range upon swelling.

### Improved determination of protein locations upon swelling

Having established that live mammalian cells and organelles can be spatially enlarged without bursting, we moved on to test if scientifically relevant information could be gained from this approach. First, we tested for improved resolution of proteins residing within membranes *vs*. lumens/matrices in organelles like the ER and mitochondria. The optical separation by light microscopy is rather impossible by diffraction-limited microscopy and difficult to accomplish by superresolution microscopy due to the close proximity of membrane and interior lumen. However, by using swollen ER and mitochondria, this task could easily be accomplished. Cells transfected with both the type 1 membrane protein CD3δ-GFP^19^ and ssRFP-KDEL as marker proteins for the ER membrane and lumen, respectively, showed upon incubation in diH_2_O unequivocally which protein is membrane-bound and which one is luminal (Fig. 4a and Supplementary Fig. S2). Equally clear results could be obtained for mitochondria, as shown by the OMM protein TOMM20-GFP and the matrix protein mCherry-Mito^16^ (Fig. 4b). In both cases, non-isotropic swelling of the organelles increased their interior volumes and also, as a consequence thereof, the distance of opposite membranes of the same tubular structure (Supplementary Videos 2, 3). As demonstrated by the corresponding plot profiles (Fig. 4a,b), the distances between the membrane of an ER tubule or a mitochondrion increased to more than 2 µm for the ER and more than 1 µm for the mitochondrion, respectively. Improved resolutions to distinguish between organelle membrane and luminal proteins became hereby feasible, even with conventional widefield microscopes (Supplementary Fig. S3).

**Figure 5:**
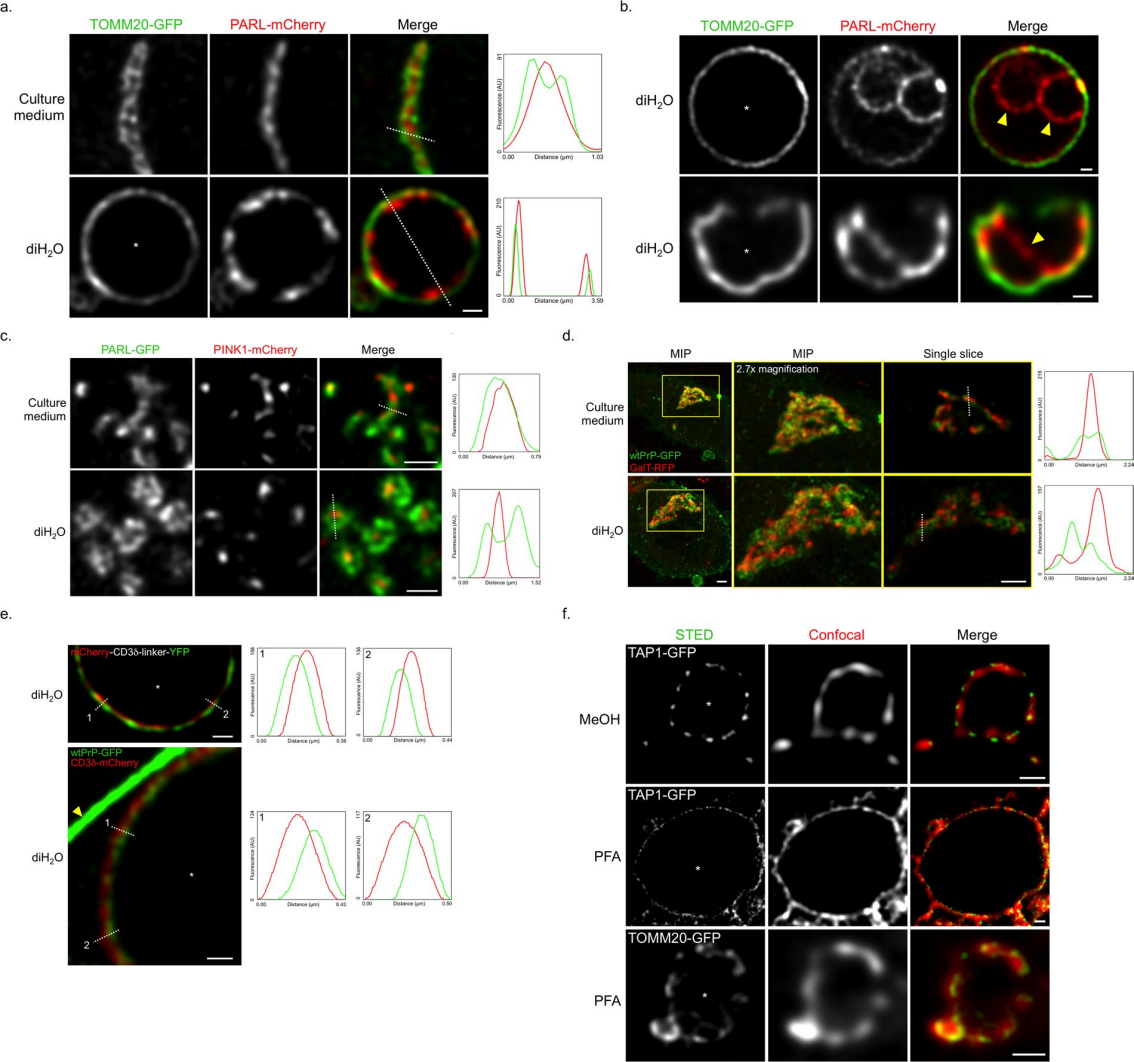
High-resolution protein location determination upon organelle swelling. (**a**) Single slice images of a COS-7 cell co-expressing TOMM20-GFP (green) and PARL-mCherry (red) pre (culture medium, top row) and post incubation in diH_2_O for 200 s (diH_2_O, bottom row). Scale bar, 0.5 µm. (**a**,**b**,**e**,**f**) The asterisks (*) (column 1) indicate the positions of mitochondrial matrix surrounded by swollen mitochondria membrane (**a**, **b** and **f**, bottom row) or ER lumen surrounded by ER membrane (**e** and **f**, rows 1 and 2). (**a**,**c**,**d**,**e**) Profiles of fluorescence intensity (arbitrary units) taken along the white dotted lines (**a**,**c**,**d**, column 3, **e**, column 1) are presented (right). (**b**) Single slice images of a COS-7 cell (top row) and a U2-OS cell (bottom row) co-expressing TOMM20-GFP (green) and PARL-mCherry (red) post incubation in diH_2_O for 200 s. The additional PARL-mCherry IMM structures are indicated (yellow arrowheads). Scale bars, 0.5 µm. (**c**) Single slice images of a HeLa cell co-expressing PARL-GFP (green) and PINK1-mCherry (red) pre (culture medium, top row) and post incubation in diH_2_O for 200 s (diH_2_O, bottom row). Scale bars, 1 µm. (**d**) MIP (columns 1 and 2) and single slice images (column 3) of a HEK cell co-expressing wtPrP-GFP (green) and GalT-RFP (red). (Columns 2 and 3) Images are 2.7x magnifications from the corresponding area outlined in column 1 (yellow rectangle). Golgi signals are shown pre (culture medium, top row) and post incubation in diH_2_O for 240 s (diH_2_O, bottom row). Scale bars, 2 µm. (**e**) Single slice images of a HeLa cell expressing (top) mCherry-CD3δ-linker-YFP or (bottom) co-expressing wtPrP-GFP (green) and CD3δ-mCherry (red) post incubation in diH_2_O for 200 s. (Top) The mCherry signal of mCherry-CD3δ-linker-YFP is shown in red and the YFP signal in green. (Bottom) For better orientation, an area of swollen ER next to the plasma membrane (yellow arrowhead) is displayed. Scale bars, 0.5 µm. (**f**) STED (green) and confocal (red) single slice images of HeLa cells co-expressing TAP1-GFP (rows 1 and 2) and TOMM20-GFP (row 3) post incubation in diH_2_O for 200 s. Shown are images of cells fixed in methanol (MeOH) (top row) or paraformaldehyde (PFA) (rows 2 and 3). Scale bars, 0.5 µm.

Next, we investigated if even more challenging protein locations could better be resolved upon incubating cells in hypotonic solutions. The mitochondrion with its complex composition of outer and inner mitochondrial membrane (IMM) and the internal matrix was perfect for this task. We transfected cells with both the aforementioned TOMM20-GFP and the IMM protein PARL, a mitochondrial rhomboid protease, tagged to mCherry (PARL-mCherry)^20^. High-resolution confocal microscopy followed by deconvolution of unaltered (i.e., untreated, pre swelling) mitochondria was, at best, hinting towards locations for TOMM20-GFP to be more outside and PARL-mCherry to be more inside of mitochondria (Fig. 5a, top row). However, even with such highly resolved images, it was impossible to determine if PARL-mCherry was located on either the IMM or the matrix. To address this, swelling of the organelle served perfectly to clarify the exact PARL-mCherry location (Fig. 5a, bottom row). By not having the PARL-mCherry signal all over the internal space, as with matrix proteins, but instead outlining the TOMM20-GFP signal from the inside, the protein’s location on the IMM could clearly be demonstrated. Surprisingly to us, imaging PARL-mCherry in swollen mitochondria revealed otherwise undetectable additional internal structures of the IMM (Fig. 5b and Supplementary Video 4) that were visible in some but not all mitochondria. To our knowledge, such structures have never been shown by light microscopy neither by diffraction-limited nor superresolution microscopy.

We also looked at constellations where two different proteins are both predicted to be on the same membrane, and tested for improved resolutions by swelling on the IMM, the Golgi and the ER. For the IMM, we imaged PARL-GFP together with mCherry-tagged PINK1^20^ (PINK1-mCherry), a kinase containing an N-terminal matrix targeting sequence followed by a transmembrane domain. When imaged before and after induction of swelling by diH_2_O, the higher resolution with swollen mitochondria led to a much clearer separation of the signals for the two proteins, which was far superior to the imaging results we got from unaltered mitochondria (Fig. 5c). For the Golgi, we tested two unrelated proteins, the aforementioned Golgi resident protein GalT-RFP and the Golgi traversing protein wtPrP-GFP^22^. The glycosylphosphatidylinositol (GPI)-anchored prion protein, PrP, is a secretory protein on its way to the plasma membrane, which upon overexpression accumulates transiently in the Golgi^23^. Again, before swelling in diH_2_O, it was not possible to distinguish the proteins’ locations within the Golgi. After swelling, the distinctly different locations for the two proteins within the Golgi could clearly be resolved (Fig. 5d and Supplementary Video 5). For the ER, we imaged proteins with FP-tags on opposite sides of the ER membrane. Two different FP constellations were tested. First, we tested a dual tag CD3δ mutant, mCherry-CD3δ-linker-YFP, with mCherry amino-terminal of CD3δ’s transmembrane domain, and YFP at the carboxyl-terminus. The protein’s correct orientation with mCherry facing the ER lumen and YFP on the cytosolic side of the ER was confirmed by fluorescence protease protection assays^19^. Second, we tested wtPrP-GFP, as GPI-anchored protein in the ER lumen, in combination with CD3δ-mCherry, as transmembrane protein with mCherry facing the cytosol. In order to keep wtPrP-GFP in the ER (or redistribute it from the Golgi), we incubated the cells in brefeldin A before swelling^24^. For both constellations, we were able to distinguish by confocal imaging which FP tag was ER luminal and which one was outside the ER. In either case, the luminal tag was clearly more prominently located on the inside of the swollen ER membrane (Fig. 5e and Supplementary Fig. S4a). The main reason for the higher resolution was the non-isotropic expansion of the ER tubules, which increased the distance of opposite sides of the same tubular membrane and consequently enabled a better distinction of proteins on the in- and outside of the ER membrane. Without swelling, the FP tags were indistinguishable in the ER (Supplementary Fig. S4b). Since all localization analyses were done with live cells, we did not even need to be concerned about, unlikely yet possible, fixation artifacts^25,26^. It is however important to add that any imaging modality that necessitates cell fixation for even higher spatial resolutions is not excluded upon cell and organelle swelling. We tested both alcohol- and aldehyde-based fixation protocols on diH_2_O-treated cells and found no detrimental effect on size and shape of the swollen cells and organelles. To demonstrate the synergistic combination of organelle swelling and superresolution microscopy with fixed cells, we performed stimulated emission depletion (STED) microscopy upon immunofluorescence labeling. Clearly higher resolutions could be achieved on the individual organelle sheaths of swollen ER and mitochondria membranes (Fig. 5f).

### Inducible and reversible swelling for higher-resolution imaging in live cells

We further explored imaging benefits, which come from employing swollen organelles in live cells. As proof of concept we demonstrate that protein dynamics on membranes can be imaged at much higher spatial resolutions, as shown by fluorescence recovery after photobleaching^27^ (FRAP) experiments of CD3δ-mCherry on individual sheaths of swollen ER (Supplementary Fig. S5 and Supplementary Video 6). The diffusion of CD3δ-mCherry on swollen ER membranes remained very similar to CD3δ-mCherry’s diffusion on the ER of untreated cells, as demonstrated by quantitative FRAP analyses (Supplementary Fig. S5b). It would be impossible to analyze individual sheaths of ER membrane in untreated cells, only whole tubules/sheets or merges thereof could be resolved with unaltered, native ER phenotypes.

By observing cells on the microscope whilst being exposed to hypotonic solutions we found diH_2_O incubation times of 1–3 min sufficient to reach cell and organelle swelling states that were useful enough for higher-resolution imaging. We wondered how the cells continued in culture after such temporary exposure to diH_2_O, and if/how they recover from this treatment. To address this, we exposed all six cell lines for 200 s to diH_2_O and observed them subsequently over time in conventional cell culture media at the microscope. All six cell lines showed no signs of elevated occurrences of cell deaths. The cells reverted to their normal shapes (i.e., flattened) soon after being replaced in culture media, and kept dividing like untreated cells (Supplementary Video 7). To further address cytotoxic effects of prolonged diH_2_O incubation, we exposed cells to even longer times in hypotonic solutions. The survival rates of all six cell lines were analyzed by incubating them in diH_2_O solutions for 5, 15, 30 and 60 min at room temperature before re-cultivation in conventional culture media. Upon diH_2_O incubation, the cells were imaged over 24 h at optimal cell culture conditions, and the survival rate was quantified from the images of 3 independent time series for each cell line and condition (Table 1). Intriguingly, most cell lines (HeLa, N2a, U-2 OS, RPE-1) showed no cell deaths and cell survival rates similar to the untreated controls of 95% or more from the population at start of the experiment, independent of the incubation time. Only COS-7 cells reacted very sensitive to prolonged diH_2_O incubation. The cells showed cell survival of less than 50% of the initial population already after 5 min in pure diH_2_O and massive cell deaths for longer diH_2_O incubation times. Also, HEK 293 cells reacted to pure diH_2_O in a way that they rounded up and detached from the well bottom, which prevented microscopy. For these two cell lines, COS-7 and HEK 293, we were able to fix both the elevated cell deaths and the detachment problem by incubating them in a 4:1 (v/v) diH_2_O/culture medium mixture. By using this mixture as hypotonic solution, which had similar swelling efficiencies, all six cell lines showed cell survival rates of 95% or more even with 60 min incubation times. Importantly, the cell did not only survive, they reverted to their normal shapes and kept dividing like untreated cells (Supplementary Video 8).

**Table 1:** Cell survival upon diH_2_O incubation. Shown are the cell survival rates of different cell lines upon incubation in diH_2_O over time (3.3, 5, 15, 30, 60 min).

Cell line	Incubation in diH_2_O prior to microscopy					
	0 min	3.3 min^#^	5 min	15 min	30 min	60 min
HeLa	+++	+++	+++	+++	+++	+++*
N2a*	+++	+++	+++	+++	+++	+++
COS-7	+++	+++	++++*	− +++*	− +++*	n.t. +++*
U-2 OS	+++	+++	+++	+++	+++	+++*
RPE-1	+++	+++	+++	+++	+++	+++*
HEK 293	+++	+++	** +++*	** +++*	** +++*	** +++*

Cell survival of the cell population is indicated: +++  is>= 95%, +is 25–50%, −is no survival. *Swelling performed with 4:1 (v/v) mixtures of diH_2_O and cell culture medium. **HEK 293 cells round up and detach, which prevents change of media prior to microscopy. n.t., not tested. All cell survival analyses for all cell lines are based on 3 independent replicates for each condition. As example, one corresponding image series for each condition is shown in Supplementary Video 8, except as indicated (^**#**^) in Supplementary Video 7.

**Figure 6:**
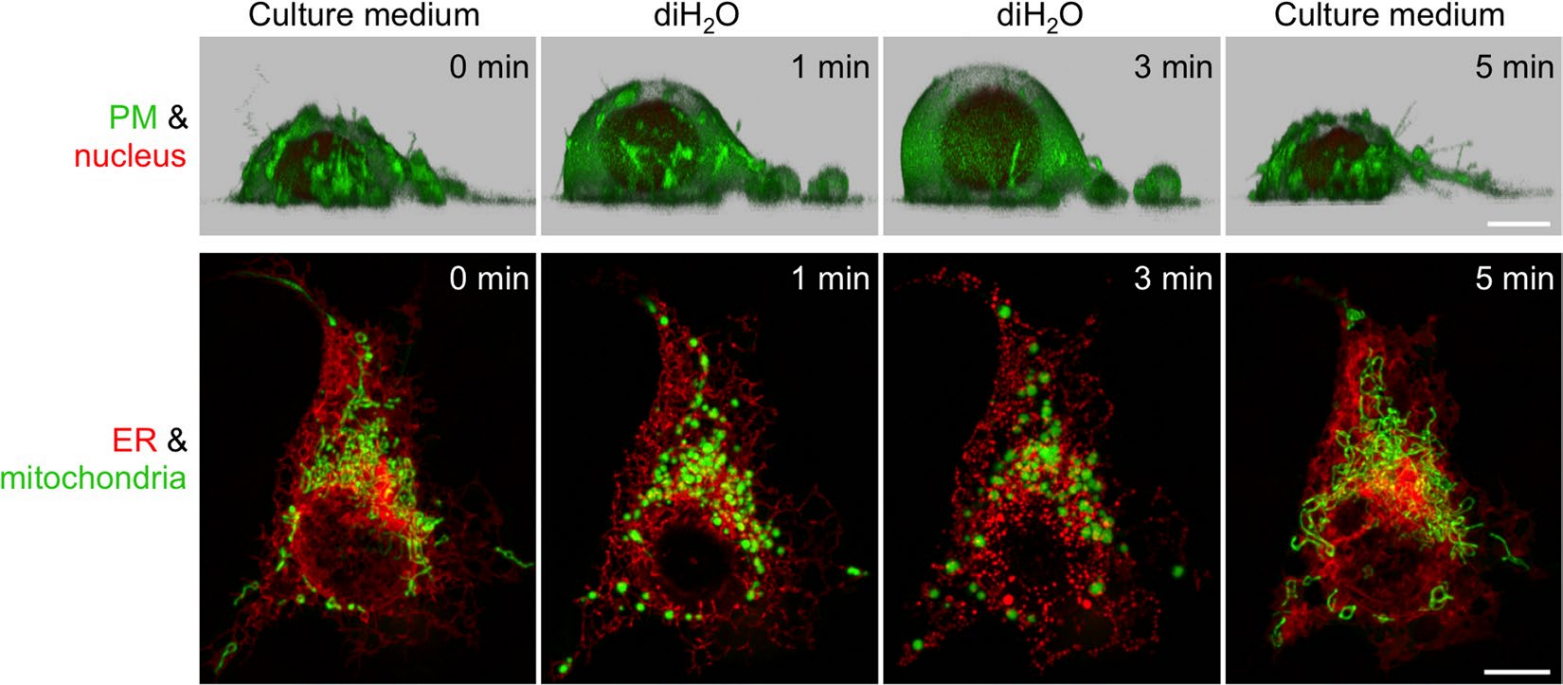
Inducible and reversible cell and organelle swelling. (Top row) Side views of 3D volume renderings of a N2a cell co-expressing the plasma membrane (PM) marker MyrPalm-mEGFP (green) and H2B-mCherry (red). (Bottom row) MIP images of a COS-7 cell co-expressing GFP-Mito (green) and ssRFP-KDEL (red). Shown are cells pre (culture medium, left column) and post incubation in diH_2_O at time points 1 min (column 2) and 3 min (column 3) and upon re-suspension in cell culture medium at 5 min (right column). Scale bars, 10 µm.

For a closer intracellular look, we imaged cells expressing fluorescent organelle marker proteins that were transiently exposed to diH_2_O, followed by re-suspension in media. Again, we were able to clearly show that the swelling of the plasma membrane, the nucleus, ER and mitochondria was inducible *and* reversible with diH_2_O and culture media, respectively, all accomplishable within a few minutes each (Fig. 6 and Supplementary Videos 9, 10). The reversibility of swelling could also be corroborated by the aforementioned whole-cell and nucleus volume increase analysis (Fig. 3a–c). Upon re-incubation in culture media, the cells and nuclei showed unswelling dynamics that were even faster compared to the volume increase upon diH_2_O incubation (Fig. 3b,c). To rule out organelle damage that could have been introduced by swelling, we also looked closer at possible ER and mitochondria fragmentation. Since swelling of the ER led to large membrane-enclosed ER units (for an example, see Supplementary Fig. S2), we tested by FRAP if the discontinuous ER upon swelling could revert to its continuous network upon re-cultivation in cell culture medium. Quantitative FRAP analyses of the luminal ER protein ssRFP-KDEL clearly showed that this was the case. The luminal protein, which stopped diffusing freely between ER units after about 5 min diH_2_O incubation, regained its free mobility throughout the whole ER upon re-cultivation of the cell in culture media (Supplementary Fig. S6).

With respect to mitochondria, we were unable to detect any fragmentation caused by swelling. High resolution imaging of single mitochondria clearly showed that these mitochondria kept their unfragmented single state and reverted to their previous size upon unswelling in cell culture media (Supplementary Fig. S7 and Supplementary Videos 10, 11).

### Improved organelle counting upon transient swelling in live cells

**Figure 7:**
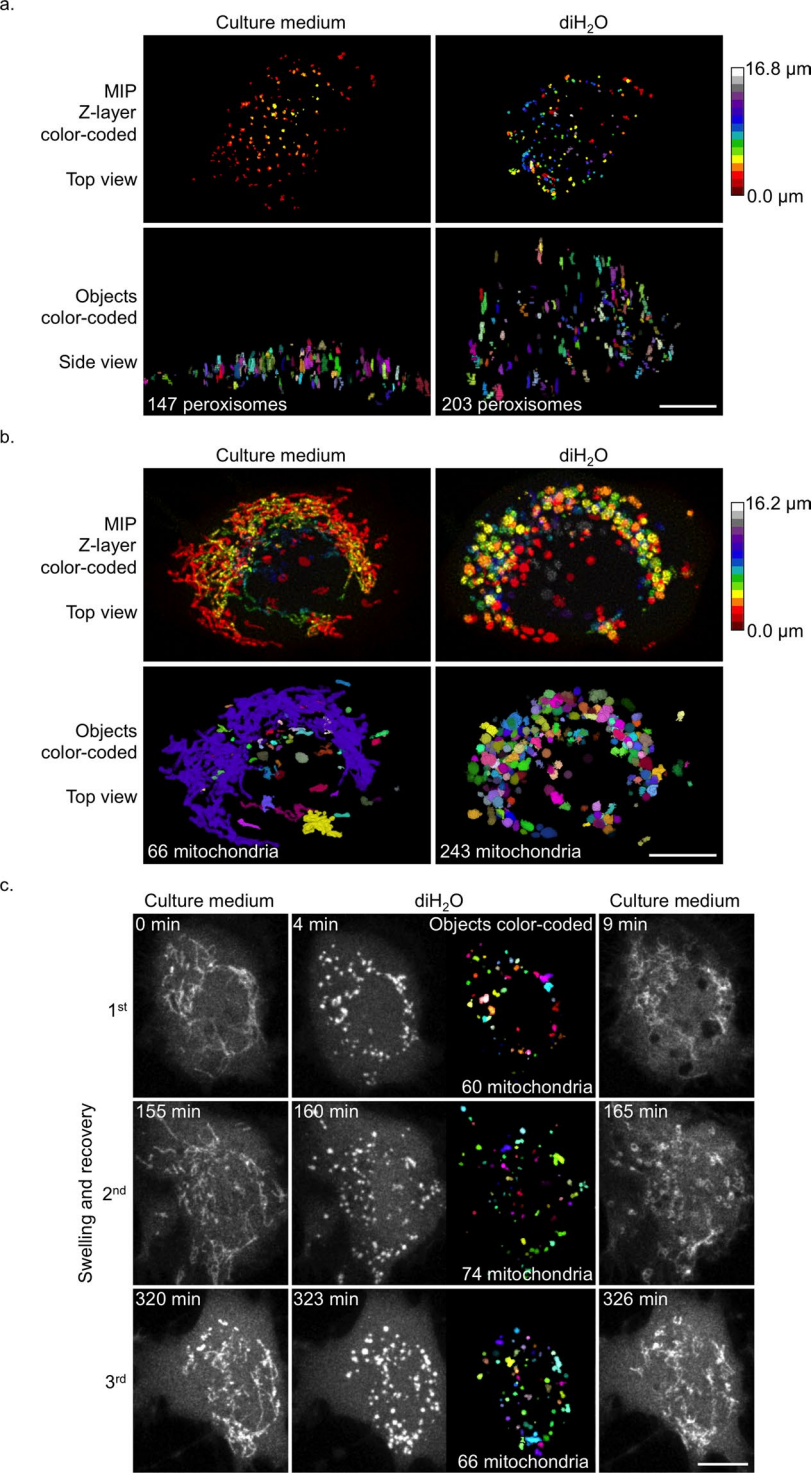
Counting of organelles upon swelling. (**a**,**b**) MIP images (top row) and corresponding (**a**) side or (**b**) top views of 3D volume renderings (bottom row) of (**a**) a RPE-1 cell expressing SKL-RFP or (**b**) a HeLa cell expressing EYFP-Mito pre (left) and post (right) incubation in diH_2_O at time point 200 s. (Top) The Z-layers of the 3D image data are color-coded. (Bottom) The individual objects are randomly color-coded. The numbers of counted (**a**) peroxisomes or (**b**) mitochondria are indicated in the bottom left corners. (**c**) MIP images of the same RPE-1 cell expressing EYFP-Mito. 3 rounds of swelling and recovery of mitochondria over a total time of 5.5 h are shown. Images were taken pre (left) and post (middle) incubation in diH_2_O and upon re-suspension in cell culture medium (right) at the indicated time points (top left corners). The individual objects are randomly color-coded (middle column on the right). The numbers of counted mitochondria are indicated in the bottom right corners (middle column). Scale bars, 10 µm.

Having established that cells recover without obvious damage from transient diH_2_O treatments, we asked if swollen cells and organelles could be utilized for further scientifically relevant analyses. We focused on resolution-dependent improvements of organelle quantifications per cell (i.e., counting organelles), since we couldn’t find any good non-invasive tools in the scientific literature. We believe there were, at least, two advantages that come with swelling to directly improve quantifications. First, the cells become significantly larger in volume, so objects like peroxisomes, intracellular vesicles or other objects freely diffusing in the cytosol have more space to redistribute and keep longer distances to each other. Second, objects that round up like mitochondria in diH_2_O could much better be segmented (i.e., individually identified) and quantified than the close-contact and intertwined native counterparts. As proof of concept we acquired 3D image stacks (Z-stacks) of cells expressing marker proteins for peroxisomes and mitochondria (Fig. 7). Indeed, the peroxisomes, which themselves do not measurably swell, occupied a much larger 3D space upon cell swelling (Supplementary Fig. S1d), thus allowing for much better quantification by image analysis. We counted before and after swelling and identified 38% more peroxisomes (pre swelling 147, post 203) for our test cell (Fig. 7a). Quantification of mitochondria was even more intriguing. Only upon swelling it became possible to quantify individual mitochondria, which in their native appearance were far too intertwined for any reasonable quantification. As rounded, swollen objects, the individual mitochondria could be resolved, segmented and counted. Image analysis revealed 243 mitochondria (+268%) in our test cell as opposed to 66, obviously wrongly counted, mitochondria that could be identified prior to swelling induction (Fig. 7b). We next tested if we could apply this analysis not only once but also in succession in live cells. We exposed the same cell to 3 consecutive rounds of both swelling and recovery over a total time of 5.5 h. The cell survived this procedure without any obvious detrimental signs, and we were able to acquire 3D image data upon swelling to quantify the number of mitochondria. We identified 60, 74 and 66 mitochondria at time points 4, 160 and 323 min respectively, demonstrating that multiple transient analyses of the same cells over time were possible (Fig. 7c). Certainly, since dynamic organelle quantifications are concerned, we do not know the ground truth with respect to absolute numbers of the organelles tested at any time of analysis. We can only rely on best possible practices. However, screening the image data by visual inspection (Supplementary Video 12), we believe to have reliably counted much closer to the true quantities and definitely much more precisely than possible without swelling.

## Discussion

We have demonstrated a variety of benefits for improved spatial resolution by induced cell and organelle swelling in hypotonic solutions. Our approach is profoundly different from all those that attempt to either keep the biosample as close as possible to unaltered cell and organelle morphologies or to enlarge it isotropically as in expansion microscopy. Our rationale to favor non-isotropic enlargements (i.e., swelling) of the sample is, in our opinion, justified. As a matter of fact, the non-isotropic expansion is exactly what makes it possible to reveal scientifically relevant information that is otherwise not (or only arduously) resolvable. Bear in mind, with fast moving and constantly shape-changing organelles like mitochondria, ER and Golgi there is anyway no such thing as two identical organelles of the same kind. Particularly not, if the sub-micrometer scale is concerned. Also, since there is no such thing as a singularly correct morphology ascertainable for dynamic organelles, any organelle at any different time point of fixation and/or live acquisition would always look different. Hence, as long as membrane and luminal protein allocations are not disturbed, information at higher resolution on protein location, dynamics and interaction can be gained upon swelling. We have tested far more membrane and luminal proteins than presented in this paper without ever finding any distorted protein locations or other noticeable problems. In comparison to expansion microscopy or any other light microscopy imaging technique, we see our approach as an additional, neither competing nor replacing, application. The strengths of expansion microscopy lie in the enlargements of tissue material like whole brains^28^, and/or those biostructures that would not profit from our approach like cytoskeleton components and centrioles^29^, even though positive de-crowding effects upon non-isotropic cell swelling might still apply. However, expansion microscopy is very time-consuming and labor-intensive, which limits any quick access to its benefits. As far as resolution is concerned, we are not aware that expansion microscopy was able to resolve, for example, distinct luminal vs. membrane protein locations in the ER (Fig. 4a and Fig. 5e), or precise protein destinations distinguishable between the IMM and OMM (Fig. 5a). Nor will it be able to reveal the additional internal structures of the IMM (Fig. 5b), as all has been presented here. Superresolution techniques like STED, PALM/STORM and SIM have successfully been employed to resolve intraorganelle protein locations, for example of mitochondria^30–33^. The investment of time, effort and instrumentation though is much higher compared to our approach, at similar scientifically relevant outcomes. It is fair to assume that neither expansion microscopy nor superresolution microscopy alone will be able to resolve intertwined organelles like mitochondria for counting purposes. This becomes only possible upon non-isotropic swelling.

Another significant difference to expansion microscopy is our approach’s applicability to live cells, which, to name two advantages, eliminates any concerns for fixation artifacts^25,26,34^ and enables high-resolution imaging of the same live sample over time. We demonstrated this here by ER membrane protein FRAP studies of an individual ER sheath (Supplementary Fig. S5 and Supplementary Video 6) and by identifying mitochondria counts in the same live cell over time (Fig. 7c). Importantly, our approach is not excluding any additional imaging modality. If the swollen state of cells and organelles is to be utilized for superresolution and/or expansion microscopy, it can be prepared as any conventional sample. That said, the very short preparation times for swelling together with the very modest optical instrumentation needs at literally no additional costs for material (chemicals etc.) makes swelling a very interesting and attractive tool for every cell researcher, including those with no immediate access to high-end (and often high-priced) optical instrumentation. The fact that it takes no more than a conventional fluorescence microscope (widefield or confocal), a drop of diH_2_O and a few minutes to find out if this approach is applicable to their project, should make it easy and appealing for any researcher to just give it a try.

## Materials and Methods

### Cell lines

All cell lines (COS-7, HEK 293, HeLa, N2a, RPE-1, U-2 OS) were obtained from the American Type Culture Collection.

### DNA constructs and reagents

The plasmids for MyrPalm-mEGFP^14^, ssRFP-KDEL^15^, GalT-RFP^17^, H2B-GFP^18^, CD3δ-GFP^19^, TOMM20-GFP^20^, PARL-GFP^20^, PINK1-mCherry^20^, TAP1-GFP^35^, TAP2-RFP^35^, wtPrP-GFP^22^ and SKL-RFP^15^ have been described previously. For CD3δ-mCherry, GFP from CD3δ-GFP was replaced by mCherry. To create mCherry-CD3δ-linker-YFP, we used YFP-CD3δ^19^ and replaced YFP with mCherry. The resulting construct was combined with CD3δ-CFP^19^ to create mCherry-CD3δ-CFP. This construct was further modified by adding YFP to its carboxyl-terminus to get mCherry-CD3δ-linker-YFP with CFP as linker sequence. For H2B-mCherry, GFP from H2B-GFP was replaced by mCherry. For pmCherry-Mito and pGFP-Mito, EYFP from pEYFP-Mito (Clontech) was replaced by mCherry and EGFP, respectively. Brefeldin A was purchased from Sigma-Aldrich and used at 5 μg/ml.

### Mammalian cell transfection

COS-7, HEK 293, HeLa, N2a and U-2 OS cells were grown in Dulbecco’s modified Eagle’s medium (DMEM, Gibco) and RPE-1 cells were grown in DMEM/F-12 nutrient mixture (Gibco), all supplemented with 10% (v/v) fetal bovine serum at 37 °C in 5% (v/v) CO_2_. Transient transfections were performed using 25 kDa linear polyethylenimine (Polysciences)^36^. 18–48 h post-transfection the cells were subjected to microscopy.

### Deionized H_2_O

For diH_2_O, an Optiprep LS purification system (membraPure GmbH, Germany) was used to get ultra pure water according to ASTM (American Society for Testing and Materials) type 1 grade and a conductivity of 0.055 µS/cm. We believe, differently produced diH_2_O will work as well. The water was refilled in smaller bottles and autoclaved before being used for cell and organelle swelling. The osmolarity of diH_2_O is 0 mOsmol/l or close to 0 mOsmol/l upon opening the bottle, respectively. The osmolarity of mammalian cells and cell culture media is 270–300 mOsmol/l. For the experiments, we either replaced the cell culture media with pure diH_2_O or we used 4:1 (v/v) diH_2_O/culture medium mixtures. The resulting osmolarities of the swelling solutions were close to 0 mOsmol/l (assuming some contamination by traces of media) or 54–60 mOsmol/l, respectively.

### Induction and reversion of cell and organelle swelling

Swelling of cells and organelles was performed at the microscope with live cells in µ-Slide 8-well dishes (ibidi). For induction, culture medium was aspirated and wells were filled with diH_2_O, either pure or as diH_2_O/culture medium mixtures. For reversion of swelling, the hypotonic solution was discarded and cells were immediately re-suspended in conditioned cell culture medium.

### Microscopy

Confocal and STED imaging was performed on a Leica TCS SP8 3X STED system with a HC PLAPO 93×/1.30 numerical aperture (NA) glycerol objective lens and a HC PLAPO 100×/1.40 NA STED White oil objective lens, or, for confocal imaging only, a HCX PLAPO 63×/1.40 NA oil objective lens (Leica Microsystems). The pinhole was set to 1 airy unit or smaller. STED images were acquired by using a white light laser at 635 nm and a STED laser of 775 nm for STAR 635 P imaging (abberior). HyD detectors (Leica Microsystems) were used for signal detection. Confocal live cell imaging was also performed on a Leica DMi8 spinning disk system equipped with a CSU-X1 scanner unit (Yokogawa), an Orca Flash 4.0 LT digital camera (Hamamatsu) and a HC PLAPO 63×/1.40 NA oil objective lens (Leica Microsystems). For fluorescence recovery after photobleaching (FRAP), a Zeiss LSM 780 microscope with a 63×/1.40 NA Plan-Apochromat oil objective lens (Zeiss) and a 561 nm diode laser was used. A small region of interest (ROI) was photobleached and fluorescence recovery was monitored over time, as described previously^37^. Widefield fluorescence microscopy was performed with an Olympus xcellence IX81 microscope system using a 60×/1.35 NA DIC UPlan-SApochromat oil objective lens (Olympus) and a XM10 digital camera (Olympus) and a dual band GFP/mCherry sbx ET filter set (Chroma). Widefield DIC microscopy was performed with an Olympus CellR IX71 microscope system using a 20×/0.5 NA UPlanFL air objective lens (Olympus) and a ProgRes MF digital camera (Olympus). For long-term widefield live cell imaging, a Nikon BioStation IM-Q system was used. Relevant specifications of all micrographs used are listed (Supplementary Table S2).

### Dye labeling of cells for STED

Transfected cells were fixed in 100% methanol at −20 °C or 3.7% paraformaldehyde (PFA) in phosphate-buffered saline (PBS) at room temperature for 10 min, and permeabilized in 0.2% Triton X-100 in PBS for 2 min. The primary antibody, anti-GFP^22^, was used for immunostaining of the ER or mitochondria proteins TAP1-GFP and TOMM20-GFP, respectively. As secondary antibody, anti-rabbit IgG labeled with the STED dye STAR 635 P (abberior) was used.

### Image processing and analysis

General image processing and final image preparations for publication were performed by using ImageJ/FIJI^38,39^. Texture-based volume renderings of image stacks were generated with the ImageJ 3D viewer^40^. For higher resolution confocal imaging (Fig. 5), all images were deconvolved using Huygens software (Scientific Volume Imaging). For organelle counting, Fiji software was used for further image analysis. Noisy images were filtered using 3D mean filter or Gaussian filter, and background subtracted. Next, the images were segmented using automatic thresholding. When required, the binary masks were further processed to eliminate small noise particles and holes. For mitochondria, the 3D Watershed Split of the 3D ImageJ Suite^21^ was applied to separate objects. Since most untreated live mitochondria are close contact, intertwined objects, 3D Watershed Split resulted in artifacts, which rendered watershed application futile for such image data. However, 3D Watershed Split could be applied to swollen mitochondria images without showing any artifacts upon visual inspection. For cell and nucleus volume measurements, we used the “MorphoLibJ”^41^ plugin to keep only the largest 3D region and remove smaller noise-induced regions. For labeling individual objects in binary images, we used the 3D Object Counter plugin^42^. A 3D shape plugin was used to measure convex volumes^43^. 3D ImageJ Suite^21^ was used to compute the 3D bounding box volume. We assigned random colors to individual objects for better visualization. For the diameter analysis, we used the “Find Peaks” command within the BAR collection^44^ to find peaks in manually marked line profiles. Full width at half maximum was computed for profiles with only one peak.

## Data Availability

All relevant data is available in the main text or the supplementary material. All plasmids, implemented software analysis tools (macros) and all image raw data are available upon request (h.lorenz@zmbh.uni-heidelberg.de).

## Supplementary information

Supplementary Video 1

Supplementary Video 2

Supplementary Video 3

Supplementary Video 4

Supplementary Video 5

Supplementary Video 6

Supplementary Video 7

Supplementary Video 8

Supplementary Video 9

Supplementary Video 10

Supplementary Video 11

Supplementary Video 12

Supplementary Information
