# Supplementary material for: Improved spatial resolution by induced live cell and organelle swelling in hypotonic solutions
Source: Sci Rep. 2019 Sep 9;9:12911. doi: 10.1038/s41598-019-49408-2 (PMC6733880; doi:10.1038/s41598-019-49408-2)
Supplement: Supplementary file 13 — Supplementary Information [file 41598_2019_49408_MOESM13_ESM.pdf]

Supplementary Information for

Improved spatial resolution by induced live cell and organelle swelling in hypotonic solutions

Astha Jaiswal, Christian H. Hoerth, Ana Mariel Zúñiga Pereira & Holger Lorenz*

*Correspondence to Holger Lorenz: h.lorenz@zmbh.uni-heidelberg.de

Application \ Imaging modality	Swelling	Expansion microscopy	STED	Localization microscopy (PALM/ STORM)	Structured illumination microscopy (SIM)	Confocal	Widefield
**Live cells**	Yes	No	Limited[1]	Very limited[2]	Yes	Yes	Yes
**Fixed samples**	Yes	Yes	Yes	Yes	Yes	Yes	Yes
**Superresolution**	Yes[3]	Yes[4]	Yes	Yes	Yes	No/ Limited[5]	No
**Large scan field 3D imaging ($\geq$ mammalian cell range)**	Yes	Yes	Limited z-range[6]	Very limited z-range[7]	Yes	Yes	Yes
**Acquisition photobleaching**	Low	Low	High	High[8]	Moderate/high	Low	Low
**Free choice of dyes, labels, fluorescent proteins**	Yes	Yes	No	No	Yes	Yes	Yes
**Effortless co-utilization with other imaging modalities for improved spatial resolution**	Yes	Yes	No	No	No	n.a.	n.a.

[1]Limited due to problems with phototoxicity and acquisition photobleaching. [2]Localization microscopy acquisition too slow for dynamic (except very slow) processes in live cells. [3]Superresolution upon non-isotropic swelling. [4]Superresolution upon isotropic expansion. [5]Only limited confocal superresolution in combination with, for example, Airyscan (Zeiss), HyVolution (Leica), IXplore SpinSR10 (Olympus). [6]Limited due to problems with acquisition photobleaching. [7]Only a few hundred nanometer with astigmatic lens or interferometry PALM. [8]Problematic with PALM applications using fluorescent proteins. n.a., not applicable.

Supplementary Table S1. **Comparison of imaging modalities.** Limitations and specific applications in light microscopy are listed (left column) and their applicability is compared among different imaging modalities.

Figure	Microscope	Imaging Modality	Objective lens	Detector	Pixel / voxel size	Pinhole airy units	Deconvolution*
2a	Olympus IX71	DIC	UPlan Fl 20x/0.50	ProgRes MF (Jenoptik)	xy323nm	n.a.	no
2b	Leica DMi8 SD	Spinning Disk	HC PL APO 63x/1.4 oil	Orca Flash 4.0 LT (Hamamatsu)	xy103nm	fixed (50µm)	no
3a (top row)	Leica TCS SP8	Confocal	HC PL APO CS2 63x/1.4 oil	Hybrid detector (Leica)	xy150nm,z300nm	1	no
3a (row 2)	Leica TCS SP8	Confocal	HC PL APO CS2 63x/1.4 oil	Hybrid detector (Leica)	xy64nm,z200nm	1	no
3a (row 3)	Leica TCS SP8	Confocal	HC PL APO CS2 93x/1.34 glyc	Hybrid detector (Leica)	xy61nm,z150nm	0.7	yes
3a (bottom row)	Leica TCS SP8	Confocal	HC PL APO CS2 63x/1.4 oil	Hybrid detector (Leica)	xy63nm,z150nm	0.75	no
3b	Leica TCS SP8	Confocal	HC PL APO CS2 63x/1.4 oil	Hybrid detector (Leica)	xy283nm,z1000nm	2	yes
3c	Leica TCS SP8	Confocal	HC PL APO CS2 63x/1.4 oil	Hybrid detector (Leica)	xy283nm,z1000nm	2	yes
3d	Leica TCS SP8	Confocal	HC PL APO CS2 63x/1.4 oil	Hybrid detector (Leica)	xy79nm.z200nm	1	yes
3e	Leica TCS SP8	Confocal	HC PL APO CS2 63x/1.4 oil	Hybrid detector (Leica)	xy79nm,z200nm	1	yes
3f	Leica TCS SP8	Confocal	HC PL APO CS2 63x/1.4 oil	Hybrid detector (Leica)	xy160nm,z1000nm	2	yes
4a	Leica TCS SP8	Confocal	HC PL APO CS2 63x/1.4 oil	Hybrid detector (Leica)	xy64nm,z300nm	1	no
4b	Leica TCS SP8	Confocal	HC PL APO CS2 93x/1.34 glyc	Hybrid detector (Leica)	xy74nm,z500nm	1	no
5a (top row)	Leica TCS SP8	Confocal	HC PL APO CS2 63x/1.4 oil	Hybrid detector (Leica)	xy54nm,z150nm	1	yes
5a (bottom row)	Leica TCS SP8	Confocal	HC PL APO CS2 63x/1.4 oil	Hybrid detector (Leica)	xy54nm,z150nm	0.75	yes
5b (top row)	Leica TCS SP8	Confocal	HC PL APO CS2 63x/1.4 oil	Hybrid detector (Leica)	xy54nm,z150nm	0.75	yes
5b (bottom row)	Leica TCS SP8	Confocal	HC PL APO CS2 63x/1.4 oil	Hybrid detector (Leica)	xy38nm,z150nm	0.6	yes
5c	Leica TCS SP8	Confocal	HC PL APO CS2 63x/1.4 oil	Hybrid detector (Leica)	xy51nm,z200nm	1	yes
5d	Leica TCS SP8	Confocal	HC PL APO CS2 63x/1.4 oil	Hybrid detector (Leica)	xy48nm,z200nm	1	yes
5e (top row)	Leica TCS SP8	Confocal	HC PL APO CS2 100x/1.40 oil	Hybrid detector (Leica)	xy21nm,z100nm	0.4	yes
5e (bottom row)	Leica TCS SP8	Confocal	HC PL APO CS2 100x/1.40 oil	Hybrid detector (Leica)	xy39nm,z100nm	0.3	yes
5f (top row)	Leica TCS SP8	STED	HC PL APO CS2 93x/1.34 glyc	Hybrid detector (Leica)	xy14nm	1	yes
5f (row 2)	Leica TCS SP8	STED	HC PL APO CS2 93x/1.34 glyc	Hybrid detector (Leica)	xy15nm	1	yes
5f (bottom row)	Leica TCS SP8	STED	HC PL APO CS2 93x/1.34 glyc	Hybrid detector (Leica)	xy15nmnm,z150nm	1	yes
6 (top row)	Leica TCS SP8	Confocal	HC PL APO CS2 63x/1.4 oil	Hybrid detector (Leica)	xy68nm,z200nm	1	no
6 (bottom row)	Leica DMi8 SD	Spinning Disk	HC PL APO 63x/1.4 oil	Orca Flash 4.0 LT (Hamamatsu)	xy103nm	fixed(50µm)	no
7a	Leica TCS SP8	Confocal	HC PL APO CS2 63x/1.4 oil	Hybrid detector (Leica)	xy65nm,z200nm	1	no
7b	Leica TCS SP8	Confocal	HC PL APO CS2 63x/1.4 oil	Hybrid detector (Leica)	xy63nm,z200nm	1	no
7c	Leica TCS SP8	Confocal	HC PL APO CS2 93x/1.34 glyc	Hybrid detector (Leica)	xy192nm,z500nm	1	no

*Huygens Professional; conf., confocal; n.a., not applicable.

Supplementary Table S2 **Image specifications.** Relevant information of all micrographs used in the main article.

Figure	Microscope	Imaging Modality	Objective lens	Detector	Pixel / voxel size	Pinhole airy units	Deconvolution*
S1a HeLa	Leica TCS SP8	Confocal	HC PL APO CS2 63x/1.4 oil	Hybrid detector (Leica)	xy52nm,z200nm	1	no
S1a HEK 293	Leica TCS SP8	Confocal	HC PL APO CS2 63x/1.4 oil	Hybrid detector (Leica)	xy48nm,z200nm	1	no
S1a N2a	Leica TCS SP8	Confocal	HC PL APO CS2 63x/1.4 oil	Hybrid detector (Leica)	xy61nm,z200nm	1	no
S1a COS-7	Leica TCS SP8	Confocal	HC PL APO CS2 63x/1.4 oil	Hybrid detector (Leica)	xy72nm,z200nm	1	no
S1a U-2 OS	Leica TCS SP8	Confocal	HC PL APO CS2 63x/1.4 oil	Hybrid detector (Leica)	xy57nm,z200nm	1	no
S1a RPE-1	Leica TCS SP8	Confocal	HC PL APO CS2 63x/1.4 oil	Hybrid detector (Leica)	xy65nm,z200nm	1	no
S1b HeLa	Leica TCS SP8	Confocal	HC PL APO CS2 63x/1.4 oil	Hybrid detector (Leica)	xy60nm,z200nm	1	no
S1b HEK 293	Leica TCS SP8	Confocal	HC PL APO CS2 63x/1.4 oil	Hybrid detector (Leica)	xy48nm,z200nm	1	no
S1b N2a	Leica TCS SP8	Confocal	HC PL APO CS2 63x/1.4 oil	Hybrid detector (Leica)	xy71nm,z200nm	1	no
S1b COS-7	Leica TCS SP8	Confocal	HC PL APO CS2 63x/1.4 oil	Hybrid detector (Leica)	xy69nm,z200nm	1	no
S1b U-2 OS	Leica TCS SP8	Confocal	HC PL APO CS2 63x/1.4 oil	Hybrid detector (Leica)	xy67nm,z200nm	1	no
S1b RPE-1	Leica TCS SP8	Confocal	HC PL APO CS2 63x/1.4 oil	Hybrid detector (Leica)	xy58nm,z200nm	1	no
S1b HeLa	Leica TCS SP8	Confocal	HC PL APO CS2 63x/1.4 oil	Hybrid detector (Leica)	xy64nm,z200nm	1	no
S1c HEK 293	Leica TCS SP8	Confocal	HC PL APO CS2 63x/1.4 oil	Hybrid detector (Leica)	xy71nm,z200nm	1	no
S1c N2a	Leica TCS SP8	Confocal	HC PL APO CS2 63x/1.4 oil	Hybrid detector (Leica)	xy68nm,z200nm	1	no
S1c COS-7	Leica TCS SP8	Confocal	HC PL APO CS2 63x/1.4 oil	Hybrid detector (Leica)	xy71nm,z200nm	1	no
S1c U-2 OS	Leica TCS SP8	Confocal	HC PL APO CS2 63x/1.4 oil	Hybrid detector (Leica)	xy69nm,z123nm	1	no
S1c RPE-1	Leica TCS SP8	Confocal	HC PL APO CS2 63x/1.4 oil	Hybrid detector (Leica)	xy69nm,z200nm	1	no
S1d HeLa	Leica TCS SP8	Confocal	HC PL APO CS2 63x/1.4 oil	Hybrid detector (Leica)	xy56nm,z200nm	1	no
S1d HEK 293	Leica TCS SP8	Confocal	HC PL APO CS2 63x/1.4 oil	Hybrid detector (Leica)	xy57nm,z200nm	1	no
S1d N2a	Leica TCS SP8	Confocal	HC PL APO CS2 63x/1.4 oil	Hybrid detector (Leica)	xy71nm,z200nm	1	no
S1d COS-7	Leica TCS SP8	Confocal	HC PL APO CS2 63x/1.4 oil	Hybrid detector (Leica)	xy64nm,z200nm	1	no
S1d U-2 OS	Leica TCS SP8	Confocal	HC PL APO CS2 63x/1.4 oil	Hybrid detector (Leica)	xy49nm,z200nm	1	no
S1d RPE-1	Leica TCS SP8	Confocal	HC PL APO CS2 63x/1.4 oil	Hybrid detector (Leica)	xy65nm,z200nm	1	no
S2	Leica TCS SP8	Confocal	HC PL APO CS2 63x/1.4 oil	Hybrid detector (Leica)	xy60nm,z150nm	0.75	no
S3	Olympus IX81	Widefield	UPLSAPO 60x/1.35 oil DIC	XM10 (Olympus)	xy108nm,,z200nm	n.a.	no
S4a	Leica TCS SP8	Confocal	HC PL APO CS2 100x/1.40 oil	Hybrid detector (Leica)	xy21nm,z100nm	0.4	yes
S4b	Leica TCS SP8	Confocal	HC PL APO CS2 100x/1.40 oil	Hybrid detector (Leica)	xy35nm,z100nm	0.5	yes
S5	Zeiss LSM 780	Confocal	PL APO 63x/1.40 oil DIC	PMT (Zeiss)	xy80nm	0.68	no
S6	Zeiss LSM 780	Confocal	PL APO 63x/1.40 oil DIC	PMT (Zeiss)	xy220nm	1	no
S7	Leica SD	Spinning Disk	HC PL APO 100x/1.4 oil	Orca Flash 4.0 LT (Hamamatsu)	xy65nm,z500nm	fixed(50µm)	no
Video1	Olympus IX71	DIC	UPlan Fl 20x/0.50	ProgRes MF (Jenoptik)	xy323nm	n.a.	no
Video2	Leica TCS SP8	Confocal	HC PL APO CS2 63x/1.4 oil	Hybrid detector (Leica)	xy64nm,z300nm	1	no
Video3	Leica TCS SP8	Confocal	HC PL APO CS2 93x/1.34 glyc	Hybrid detector (Leica)	xy74nm,z500nm	1	no
Video4	Leica TCS SP8	Confocal	HC PL APO CS2 63x/1.4 oil	Hybrid detector (Leica)	xy38nm,z150nm	0.6	yes
Video5	Leica TCS SP8	Confocal	HC PL APO CS2 63x/1.4 oil	Hybrid detector (Leica)	xy48nm,z200nm	1	yes
Video6	Zeiss LSM 780	Confocal	PL APO 63x/1.40 oil DIC	PMT (Zeiss)	xy80nm	0.68	no
Videos7 and 8	Nikon Biostation	Widefield	20x/0.8 air	CCD camera (Nikon)	xy323nm	n.a.	no
Video9	Leica SD	Spinning Disk	HC PL APO 63x/1.4 oil	Orca Flash 4.0 LT (Hamamatsu)	xy103nm,z500nm	fixed(50µm)	no
Video10 COS-7	Leica TCS SP8	Confocal	HC PL APO CS2 63x/1.4 oil	Hybrid detector (Leica)	xy102nm,z300nm	1	no
Video10 N2a	Leica TCS SP8	Confocal	HC PL APO CS2 63x/1.4 oil	Hybrid detector (Leica)	xy51nm,z300nm	1	no
Video10 RPE-1	Leica TCS SP8	Confocal	HC PL APO CS2 63x/1.4 oil	Hybrid detector (Leica)	xy52nm,z300nm	1	no
Video10 U-2 OS	Leica TCS SP8	Confocal	HC PL APO CS2 63x/1.4 oil	Hybrid detector (Leica)	xy141nm,z300nm	1	no
Video11	Leica SD	Spinning Disk	HC PL APO 100x/1.4 oil	Orca Flash 4.0 LT (Hamamatsu)	xy65nm,z500nm	fixed(50µm)	no
Video12	Leica TCS SP8	Confocal	HC PL APO CS2 63x/1.4 oil	Hybrid detector (Leica)	xy63nm,z200nm	1	no

*Huygens Professional; conf., confocal; n.a., not applicable.

Supplementary Table S2 *cont*. **Image specifications.** Relevant information of all micrographs used in the supplementary information and videos.

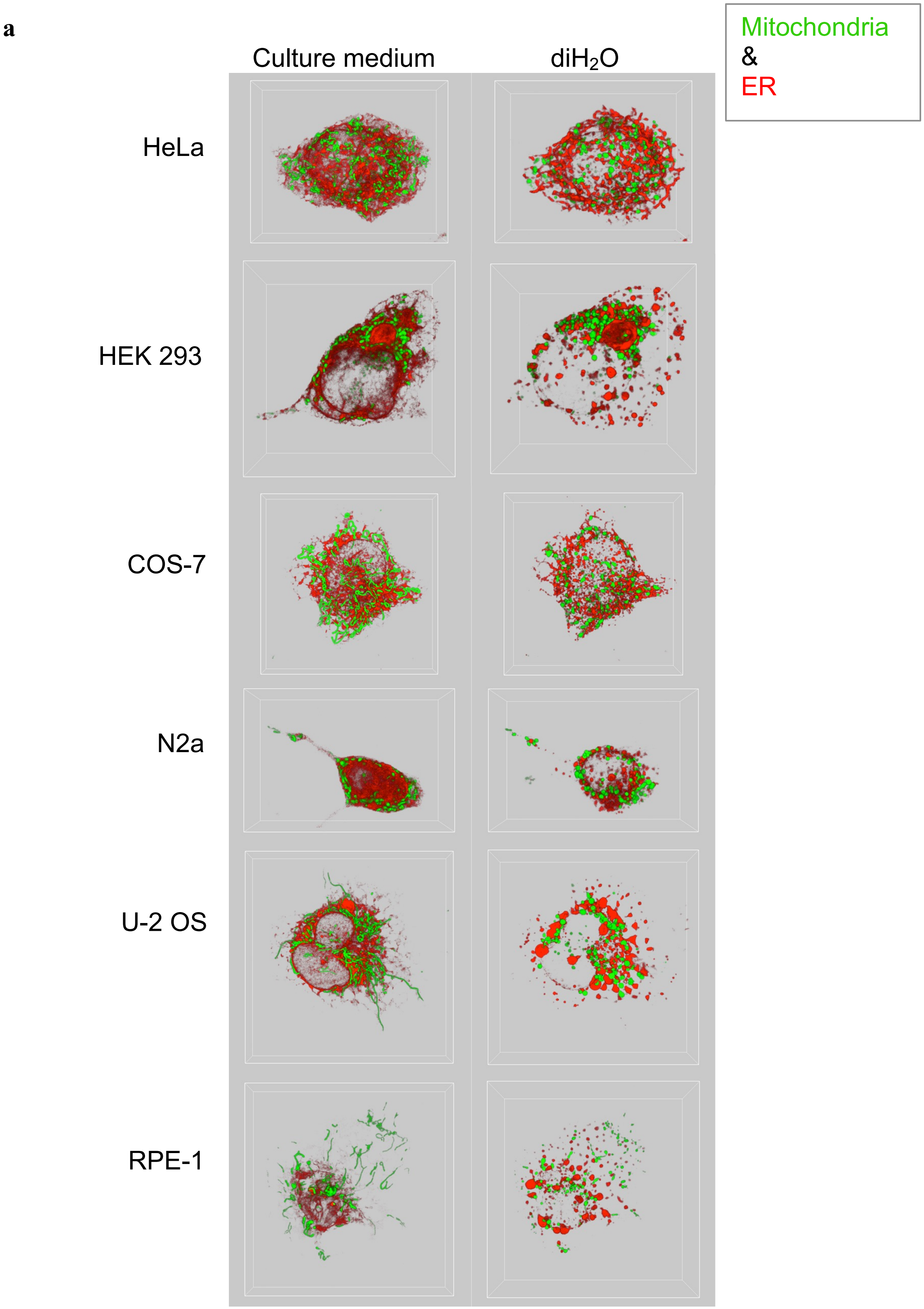

Supplementary Fig. S1. **Induced cell and organelle swelling. (a)** Top views of 3D volume renderings of cells co-expressing GFP-Mito (green) and ssRFP-KDEL (red).

b

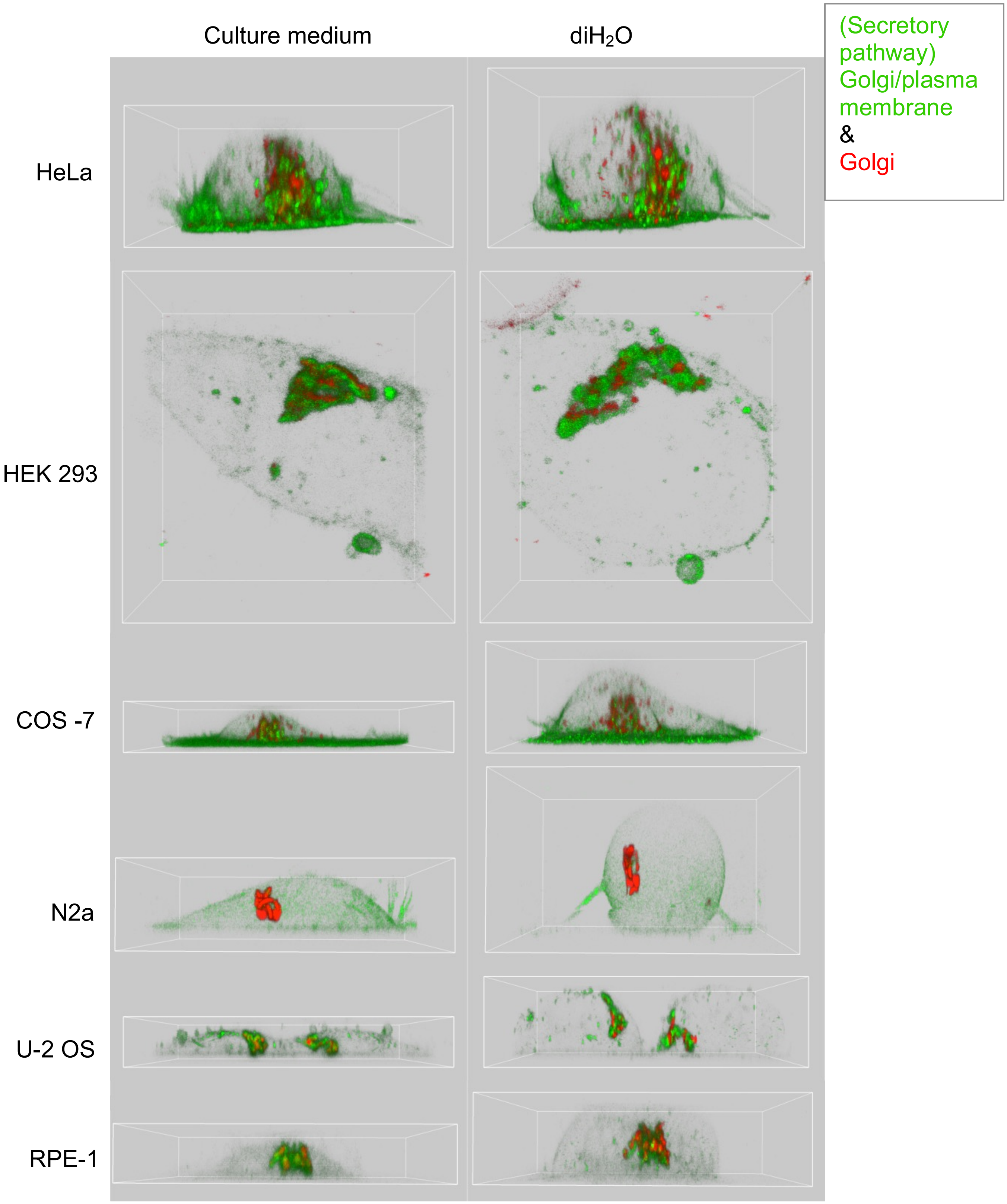

Supplementary Fig. S1 *cont.* **(b)** Side views (except HEK 293 cell with top views) of 3D volume renderings of cells co-expressing wtPrP-GFP (green) and GalT-RFP (red).

c

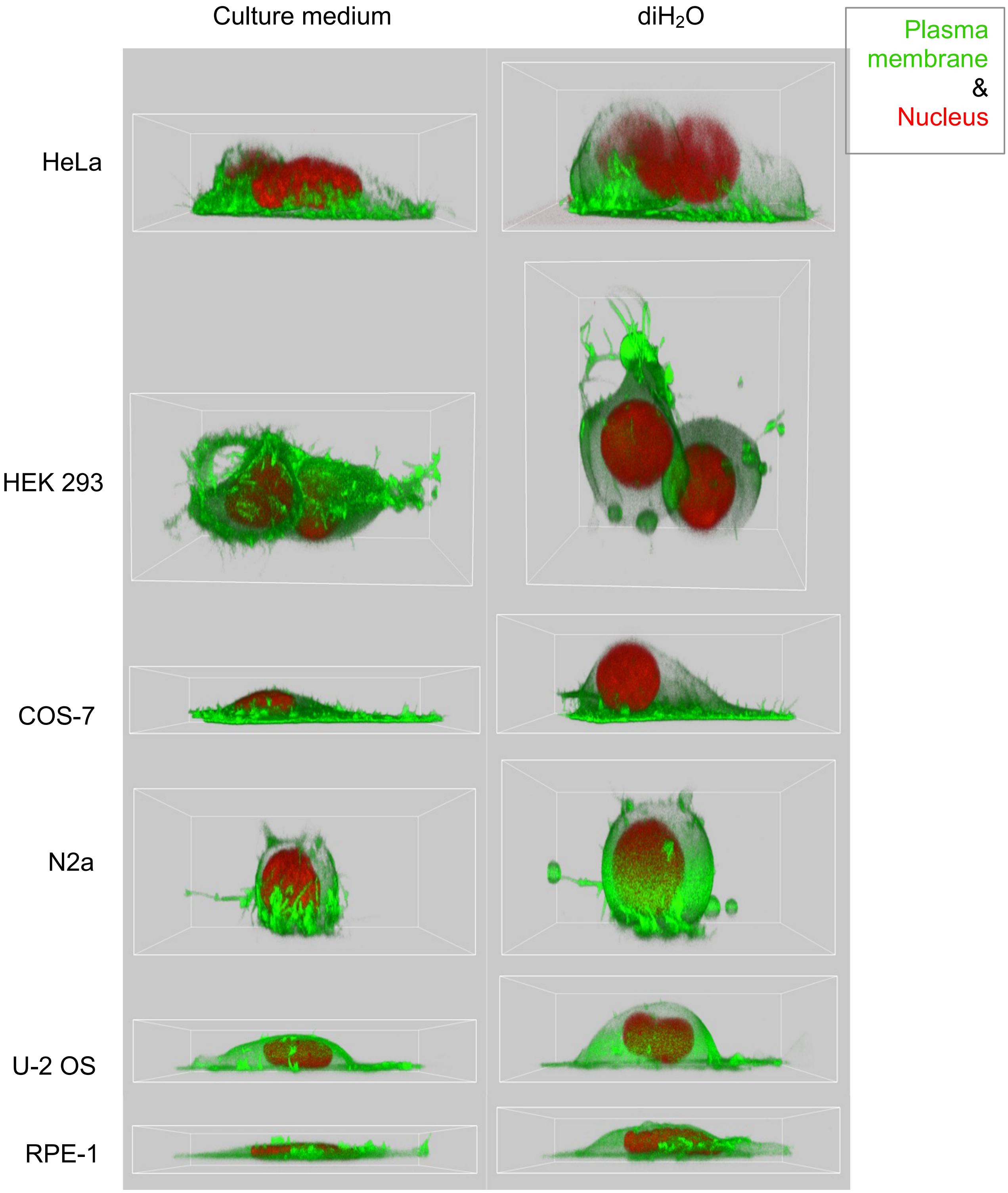

Supplementary Fig. S1 *cont.* **(c)** Side views of 3D volume renderings of cells co-expressing MyrPalm-mEGFP (green) and H2B-mCherry (red).

d

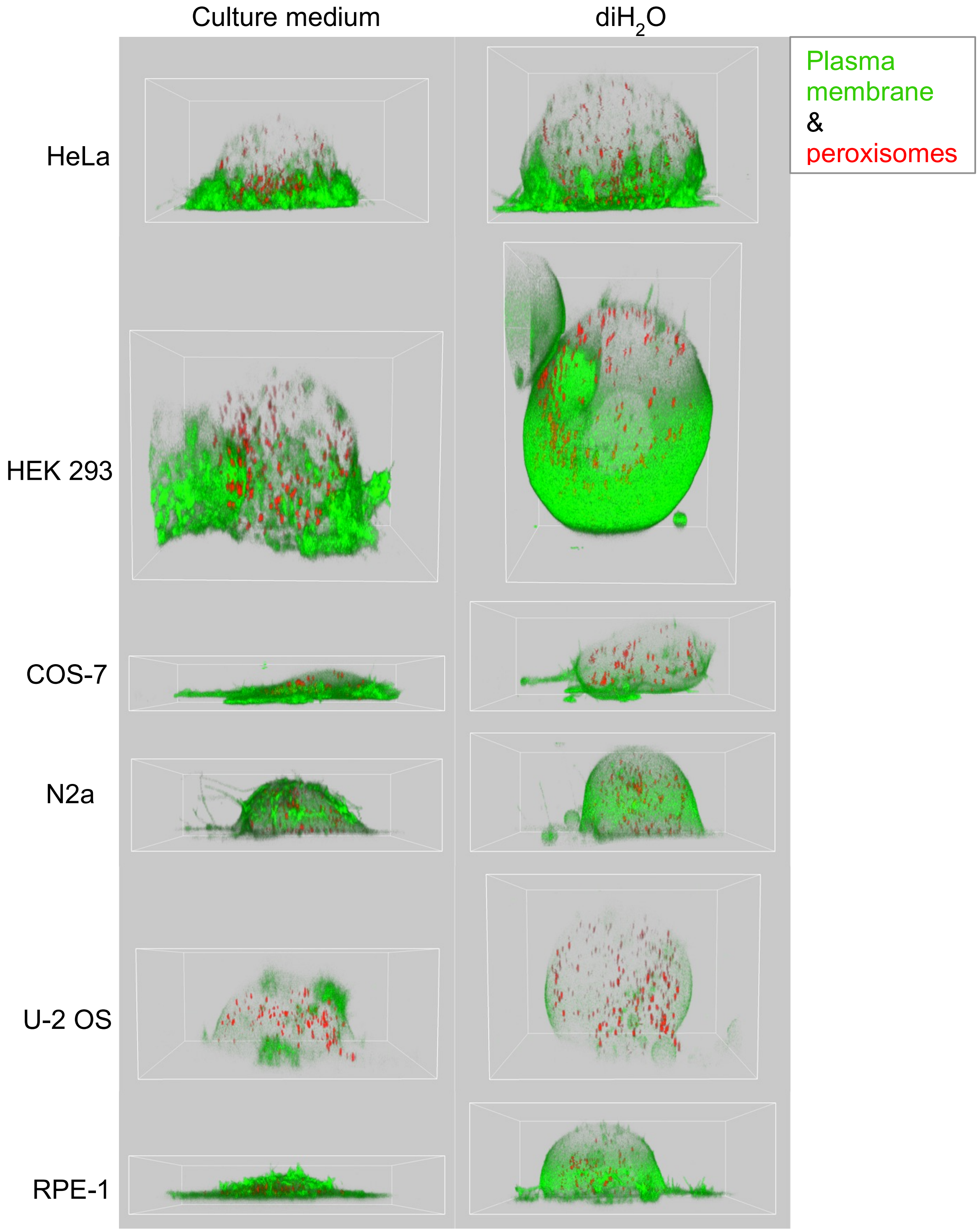

Supplementary Fig. S1 *cont.* **(d)** Side views of 3D volume renderings of cells co-expressing MyrPalm-mEGFP (green) and SKL-RFP (red). **(a, b, c, d)** Shown is the same cell for each cell line pre (culture medium, left column) and post incubation in diH_2O at time point 180 s ± 60 s (right column). Each cell is shown at identical scale for both conditions.

e

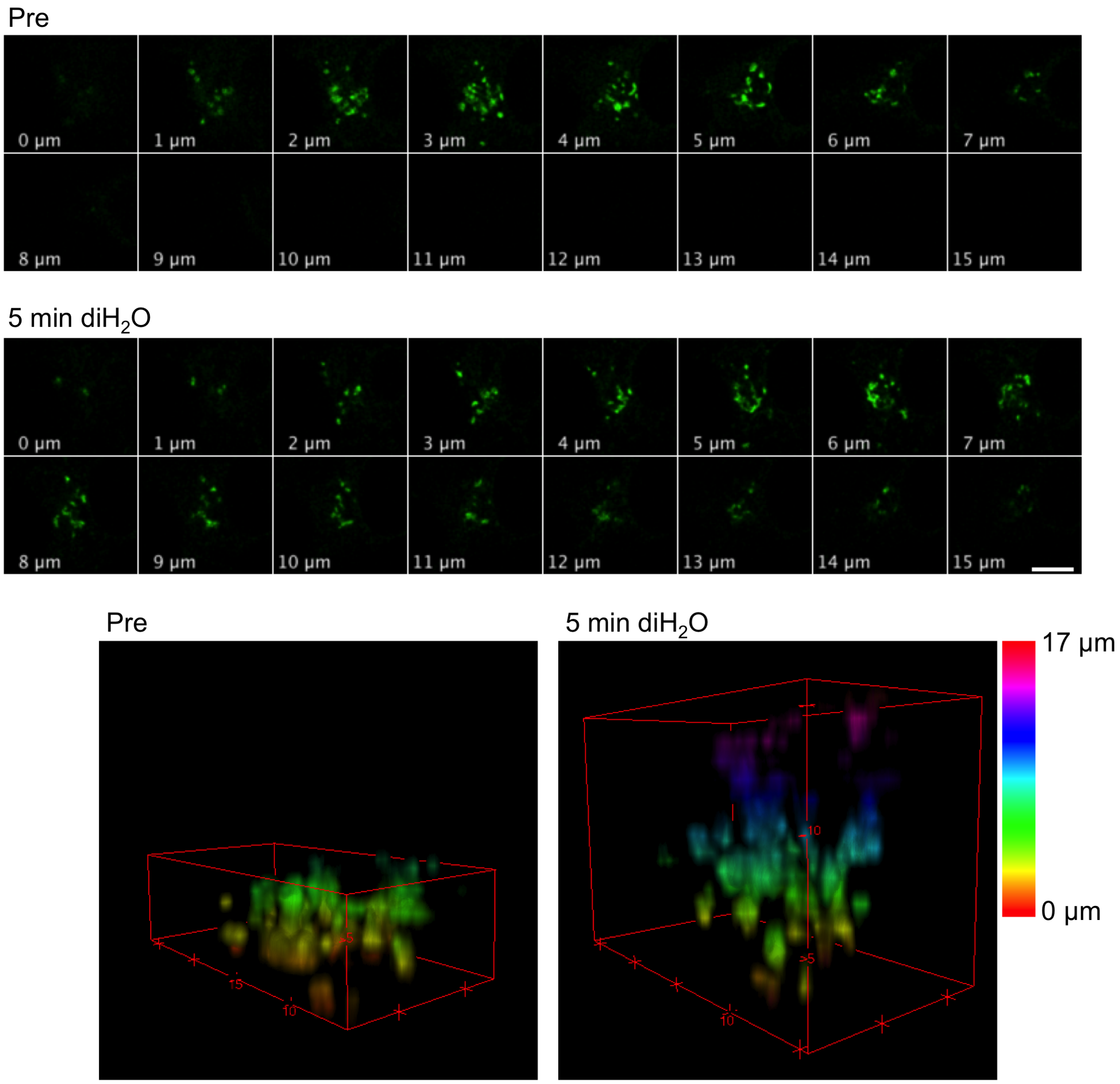

Supplementary Fig. S1 *cont.* **(e)** Measurement of 3D Golgi shape and spread upon incubation in diH_2O. Z-stacks of, here as an example, COS-7 cells expressing the Golgi marker GalT-RFP were acquired pre (top panel) and post incubation in diH_2O (middle panel, 5 min diH_2O). The Golgi signal for each condition was segmented and used to determine the smallest 3D bounding box (bottom panel, red boxes) for the pre (left) and post condition (right, 5 min diH_2O) in order to indicate the increased size and spread of the Golgi in 3D, as analyzed in Fig. 3 f in the main article. Here, the Z-layers of the 3D image data from the segmented Golgi signal are color-coded. Scale bar, 10 µm.

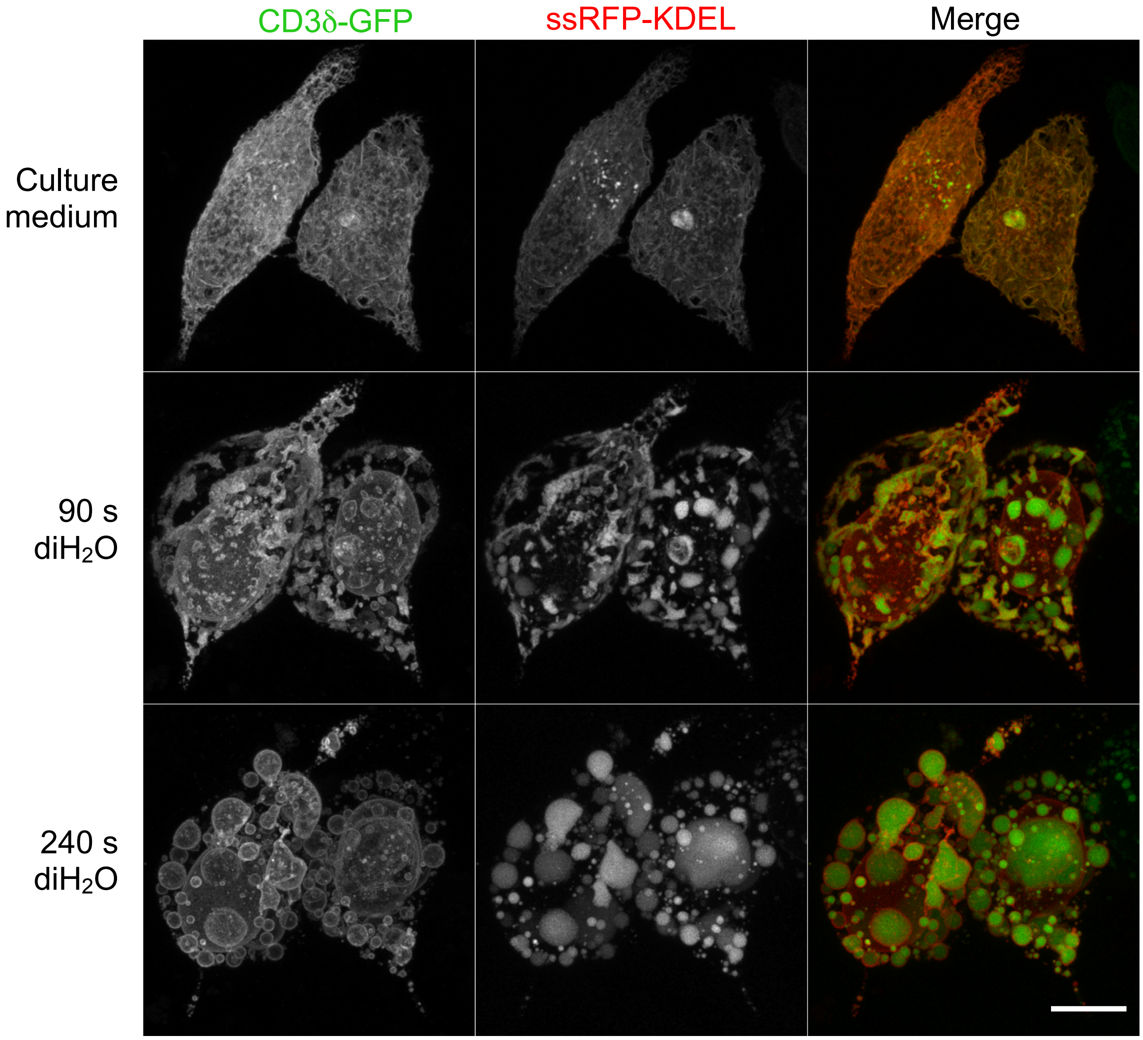

Supplementary Fig. S2. **Swelling of ER by expansion of ER tubular membranes in hypotonic solutions.** MIP images of HeLa cells co-expressing CD3δ-GFP (green) and ssRFP-KDEL (red) pre (culture medium, top row) and post incubation in diH_2O at time points 90 s (row 2) and 240 s (row 3). Similar states of swollen ER were used in Fig. 5 (main article) and in Supplementary Fig. S5. Scale bar, 10 μm.

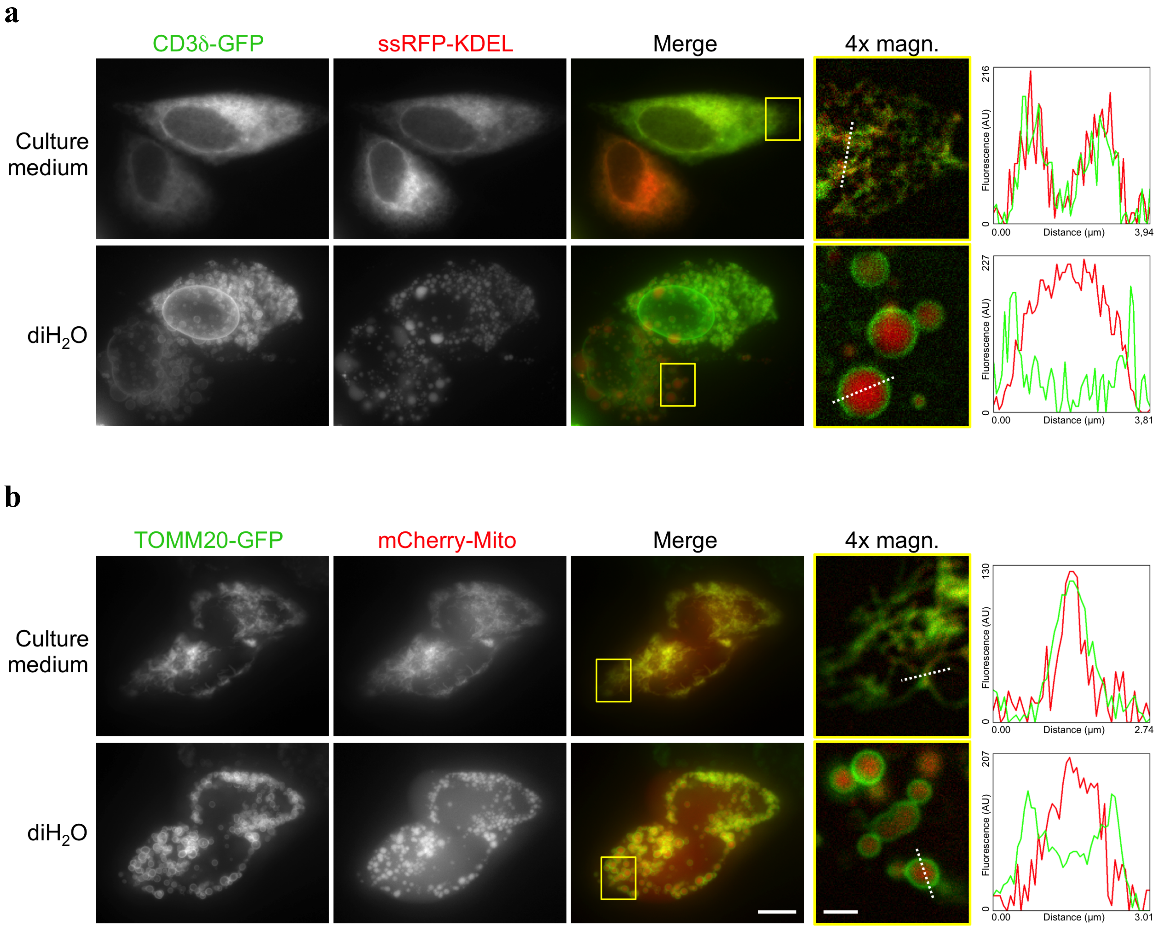

Supplementary Fig. S3. **Improved protein location determination upon organelle swelling by using widefield fluorescence microscopy. (a, b)** MIP images (columns 1-3) and single slice images (column 4) of **(a)** HeLa cells co-expressing CD3δ-GFP (green) and ssRFP-KDEL (red) and **(b)** U-2 OS cells co-expressing TOMM20-GFP (green) and mCherry-Mito (red). The single slice images (column 4) are 4.0x magnifications (magn.) of the corresponding area outlined in column 3 (merge, yellow rectangle). Profiles of fluorescence intensity (arbitrary units) taken along the white dotted lines (column 4) are presented (right). ER **(a)** and mitochondria **(b)** signals are shown pre (culture medium, top rows) and post incubation in diH$_2$O for 200 s (diH$_2$O, bottom rows). Scale bars, 10 µm (column 3), 2 µm (column 4).

a

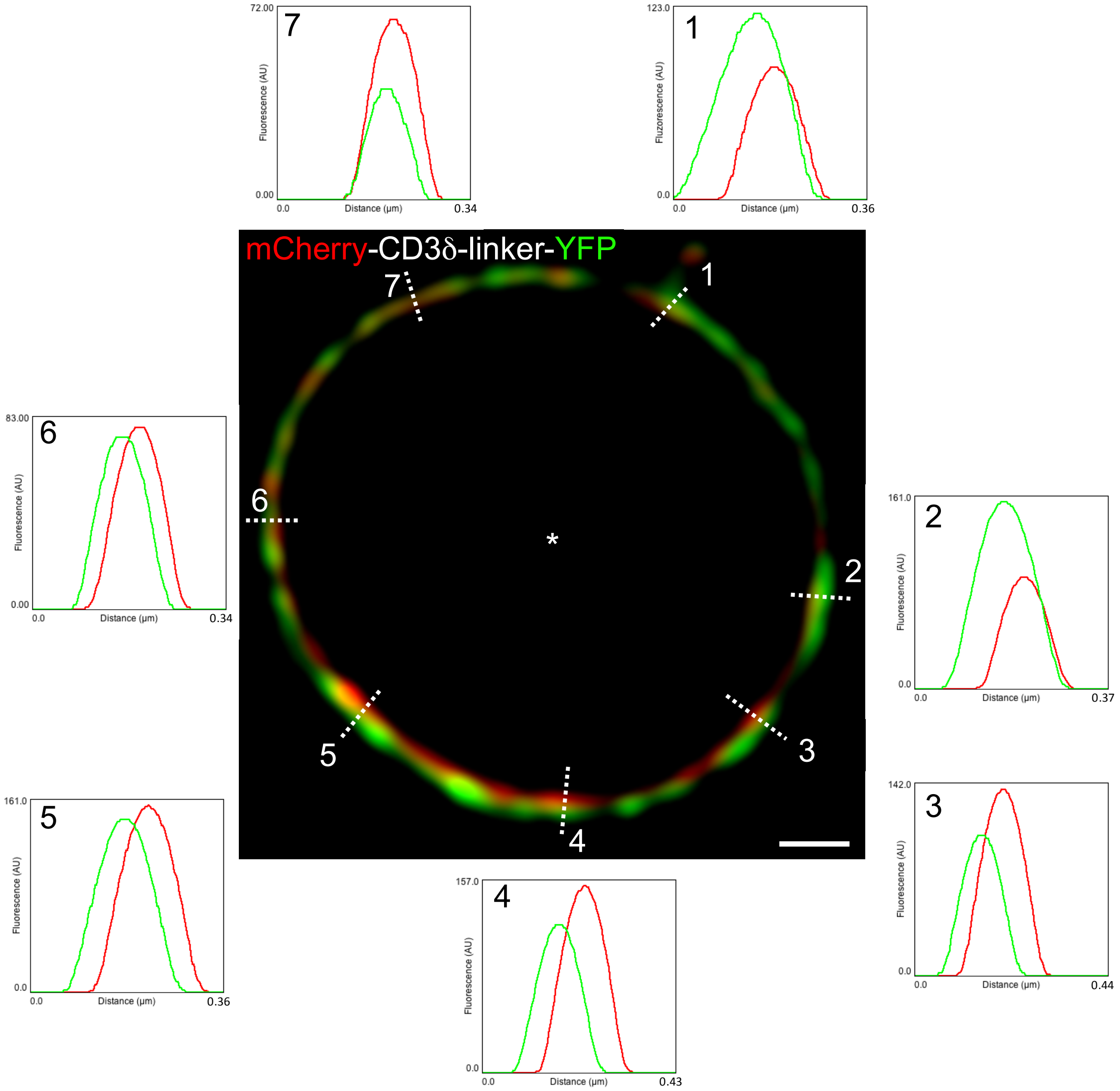

Supplementary Fig. S4. **High-resolution protein location determination upon ER swelling. (a)** Single slice image of a swollen ER tubule of a HeLa cell expressing mCherry-CD3δ-linker-YFP post incubation in diH_2O for 200 s. The mCherry signal of mCherry-CD3δ-linker-YFP is shown in red and the YFP signal in green. The asterisk (*) indicates the position of ER lumen surrounded by ER membrane. Profiles of fluorescence intensity (arbitrary units) taken along the white dotted lines and always drawn from outside to inside the ER are presented. Numbering of profiles corresponds to the numbering of dotted lines. Profiles demonstrate mCherry signal to be further inside the ER tubule than the YFP signal. The image corresponds to Fig. 5 e in the main article, here showing the whole swollen ER tubule. Scale bar, 0.5 μm.

b

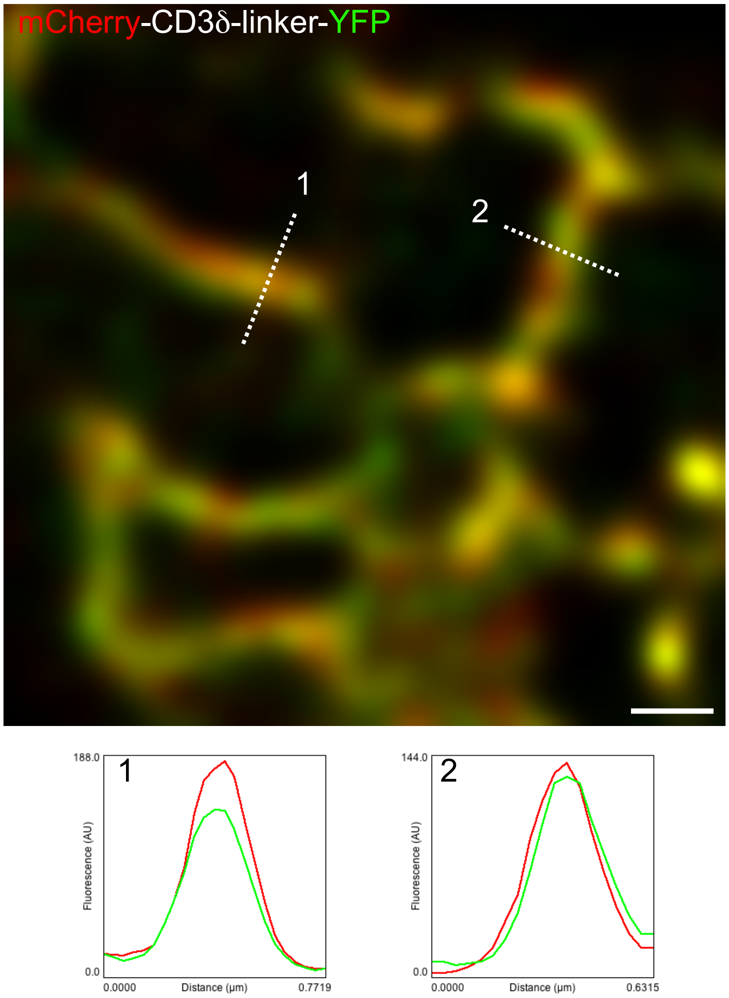

Supplementary Fig. S4 *cont.*

(b) Single slice image of an ER region of a HeLa cell expressing mCherry-CD3δ-linker-YFP in cell culture medium. The mCherry signal of mCherry-CD3δ-linker-YFP is shown in red and the YFP signal in green. Profiles of fluorescence intensity (arbitrary units) taken along the white dotted lines are presented. Numbering of profiles corresponds to the numbering of dotted lines. Profiles demonstrate that mCherry and YFP signals are indistinguishable in ER tubules without swelling. Scale bar, 0.5 μm.

a

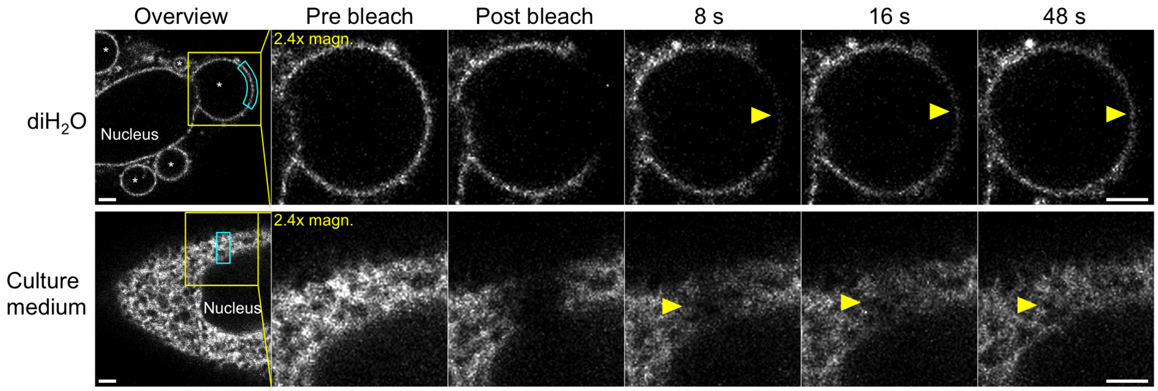

b

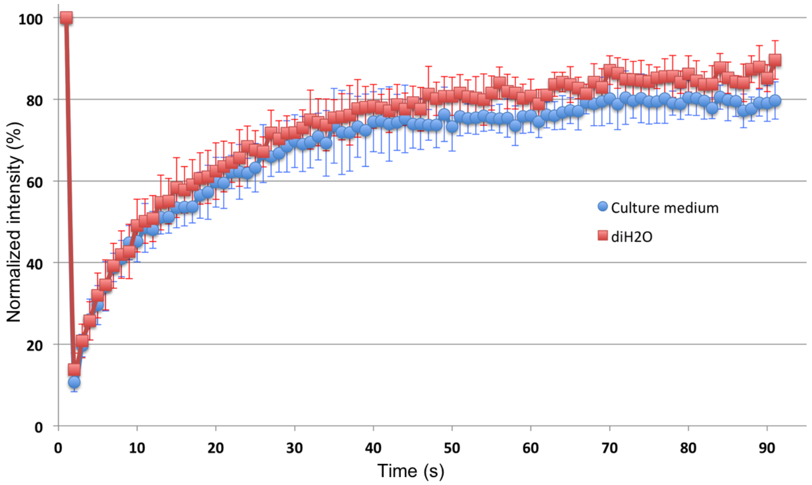

Supplementary Fig. S5. **High resolution imaging of ER membrane protein dynamics on individual ER sheath. (a)** Single slice images of a region of ER of a HeLa cell expressing CD3δ-mCherry upon incubation in diH_2O (top) or in cell culture medium (bottom). Shown are FRAP experiments with images before (pre bleach) and after photobleaching at time points 0 s (post bleach), 8 s, 16 s and 48 s of the area outlined (cyan box). The images (columns 2-6) are 2.4x magnifications (magn.) of the corresponding area outlined in column 1 (overview, yellow rectangle). For orientation, the position of the nucleus is indicated (nucleus). The asterisks (*) indicate positions of ER lumen surrounded by ER membrane (column 1, top row). Recovery of signal within the ER membrane (yellow arrowheads) can be observed at much higher spatial resolution upon swelling since membrane on opposite sides of the same tubule is wider apart. Please also see corresponding Supplementary Video 6 for better display of CD3δ-mCherry dynamics. Scale bars, 2 µm. **(b)** FRAP analysis of HeLa cells expressing CD3δ-mCherry upon incubation in diH_2O (red squares) or in cell culture medium (blue circles). Shown are mean recovery curves over 90 s upon photobleaching (at 0 s) ± standard deviations of the ER from 6 cells for each condition.

a

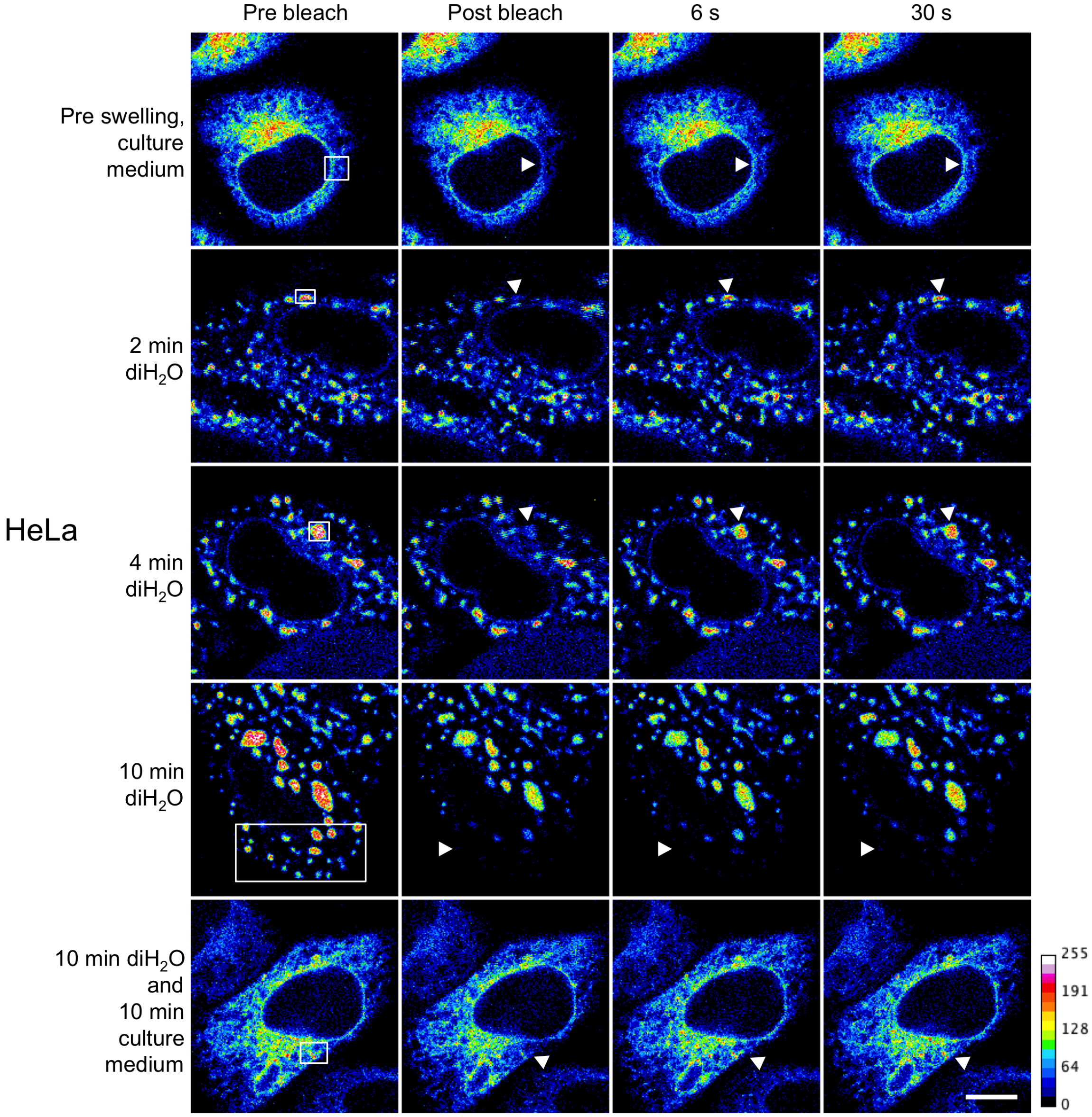

Supplementary Fig. S6. **ER fragmentation upon prolonged diH_2O incubation and ER recovery upon re-cultivation in culture medium. (a)** Single slice images of the ER of HeLa cells expressing the luminal ER protein ssRFP-KDEL in cell culture medium (top row) or in diH_2O (row 2-4) or in diH_2O followed by culture medium (bottom row) for the indicated times. Shown are FRAP experiments with images before (pre bleach) and after photobleaching (post bleach) of the area outlined (white box) and at 6 s and 30 s. Recovery areas are indicated (white arrows). Scale bar, 10 µm.

b

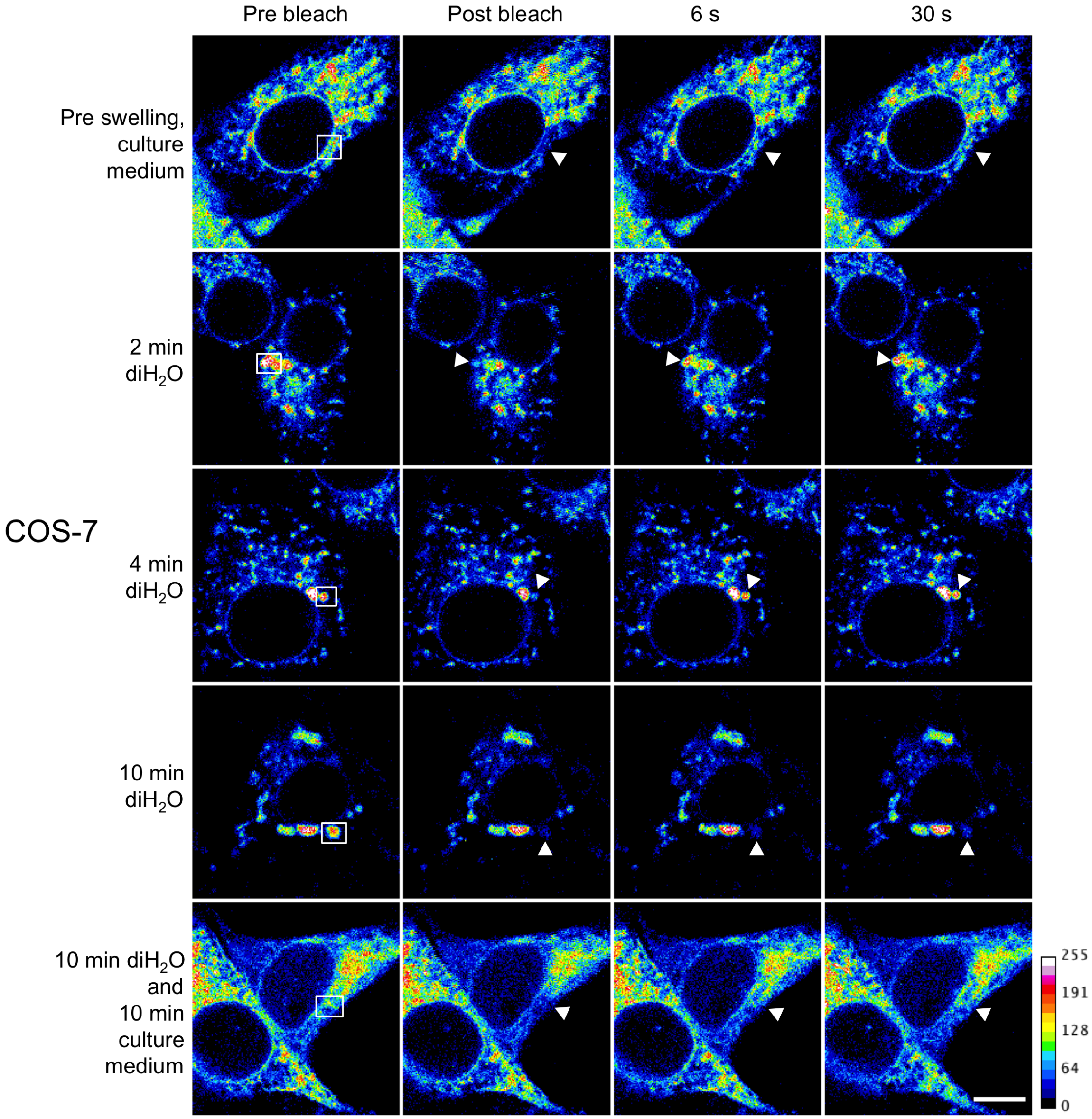

Supplementary Fig. S6 *cont.*

(b) Single slice images of the ER of COS-7 cells expressing the luminal ER protein ssRFP-KDEL in cell culture medium (top row) or in diH_2O (row 2-4) or in diH_2O followed by culture medium (bottom row) for the indicated times. Shown are FRAP experiments with images before (pre bleach) and after photobleaching (post bleach) of the area outlined (white box) and at 6 s and 30 s. Recovery areas are indicated (white arrows). Scale bar, 10 µm.

c

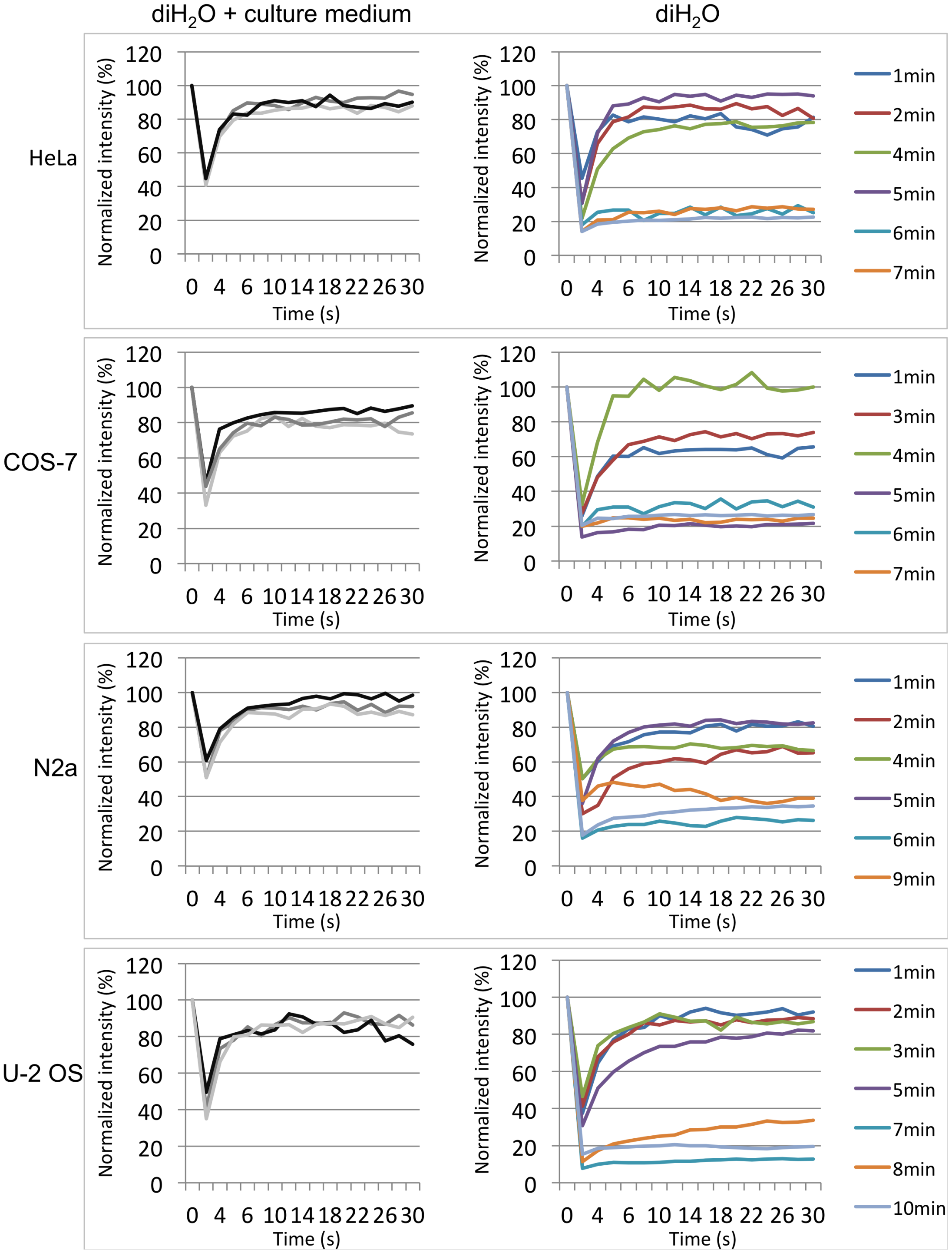

Supplementary Fig. S6 *cont.*

(c) FRAP analysis of ssRFP-KDEL mobile fractions upon incubation of cells in diH_2O for 10 min followed by incubation in culture medium for 10 min (left column), or in diH_2O (right column) for the indicated times (colored lines). Shown are recovery curves over 30 s from individual experiments upon photobleaching (at 0 s) of the ER. Each recovery curve comes from a different cell upon incubation in diH_2O as indicated.

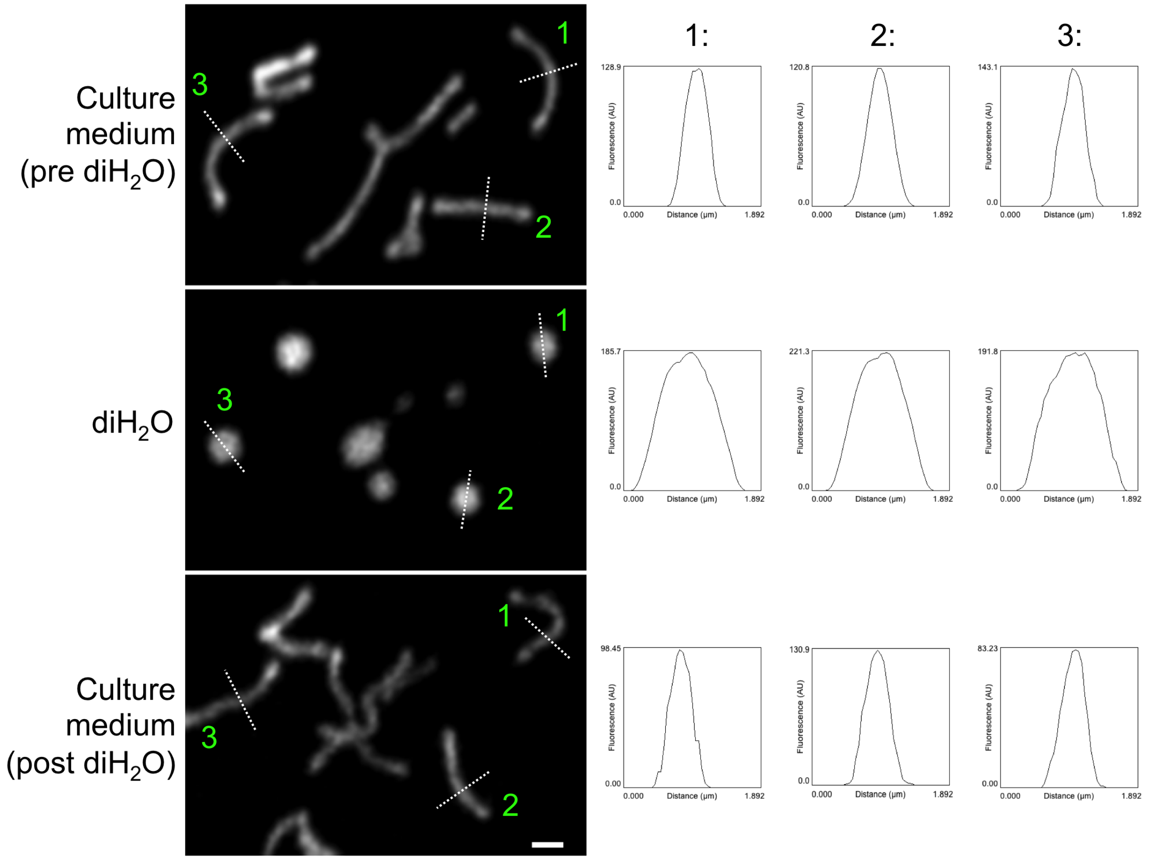

Supplementary Fig. S7. **No mitochondria fragmentation upon diH$_2$O incubation and recovery to initial mitochondria size upon re-cultivation in cell culture medium.** MIP images of mitochondria of a U-2 OS cell expressing mCherry-Mito. Shown are the identical mitochondria before (top), whilst (middle) and after (bottom) diH$_2$O incubation. Profiles of fluorescence intensity (arbitrary units) taken along the white dotted lines are presented (right) for 3 individual mitochondria and for each time point. Scale bar, 1 µm. Please also see the corresponding Supplementary Video 11 for better display of mitochondria movement, swelling and unswelling.

Supplementary Video legends

Supplementary Video 1
DIC image series of live (top row) HeLa (left), N2a (middle) and COS-7 (right) cells and (bottom row) U-2 OS (left), RPE-1 (middle) and HEK-293 (right) cells upon incubation in diH_2O over 256 s. The movie starts immediately after diH_2O was applied to the cells.

Supplementary Video 2
Time series over 580 s of MIP images (top row) and corresponding top (middle row) and side (bottom row) views of 3D volume renderings of a U-2 OS cell co-expressing CD3δ-GFP (green) and ssRFP-KDEL (red) pre and post incubation in diH_2O. DiH_2O was applied to the cells at time point 20 s. The movie corresponds to Fig. 4 a in the main article.

Supplementary Video 3
MIP image series over 252 s of a COS-7 cell co-expressing TOMM20-GFP (green) and mCherry-Mito (red) pre and post incubation in diH_2O. DiH_2O was applied to the cells at time point 10 s. The movie corresponds to Fig. 4 b in the main article.

Supplementary Video 4
Series of confocal images along the axial direction (top row) and corresponding top views of 3D volume renderings (bottom row) of a mitochondrion of a U-2 OS cell co-expressing TOMM20-GFP (green) and PARL-mCherry (red) post incubation in diH_2O for 200 s. The movie corresponds to Fig. 5 b in the main article.

Supplementary Video 5
Series of confocal images along the axial direction (top row) of a Golgi apparatus of a HEK-293 cell co-expressing wtPrP-GFP (green) and GalT-RFP (red) pre (top) and post incubation in diH_2O for 240 s. The movie corresponds to Fig. 5 d in the main article.

Supplementary Video 6
FRAP experiments of ER regions of HeLa cells expressing CD3δ-mCherry upon incubation in diH_2O (top) or in cell culture medium (bottom). The higher spatial resolution of recovery of CD3δ-mCherry signal (yellow arrowheads) within an individual ER sheath (top, right) as opposed to close contact, overlapping ER tubules (bottom, right) is shown at 2.4x magnification (right). The movie corresponds to Supplementary Fig. S5.

Supplementary Video 7
Long-term (23 h) brightfield image series of live (top row) HeLa (left), N2a (middle) and COS-7 (right) cells and (bottom row) U-2 OS (left), RPE-1 (middle) and HEK-293 (right) cells post incubation in diH_2O for 200 s. The movies start immediately after diH_2O was replaced by cell culture medium.

Supplementary Video legends *cont.*

Supplementary Video 8
Long-term (24 h) brightfield image series of live (top to bottom row) HeLa, N2a, COS-7, U-2 OS, RPE-1 and HEK-293 cells post incubation in diH_2O for 0, 5, 15, 30 and 60 min. The movies start immediately after diH_2O was replaced by cell culture medium. The movie corresponds to Table 1 in the main article. Only movies of cells with high survival rates are shown that were incubated in either pure diH_2O or, when appropriate, in a 4:1 (v/v) diH_2O/culture medium mixture (*).

Supplementary Video 9
Series of MIP images of a COS-7 cell expressing GFP-Mito (green) and ssRFP-KDEL (red). Shown are cells pre (0 min) and post incubation in diH_2O at time points 1-3 min and upon re-suspension in cell culture medium from 5-30 min. The movie corresponds to Fig. 6 in the main article.

Supplementary Video 10
Series of MIP images of (top to bottom) COS-7, N2a, RPE-1 and U-2 OS cells expressing TOMM20-GFP (green) and mCherry-Mito (red) pre (0 s) and post (from 15 s) incubation in diH_2O and upon re-suspension in cell culture medium (from 210 s) over a time course of 8 min.

Supplementary Video 11
MIP image time series of mitochondria from a U-2 OS cell expressing mCherry-Mito. The hypotonic solution was added at time point 20 s and replaced by culture medium at time point 150 s. Image series to demonstrate the recovery of diH_2O-induced swollen mitochondria to their normal sizes upon re-cultivation in cell culture medium. Please note that no fragmentation is induced upon swelling, and that the mitochondria show normal movement after unswelling. The movie corresponds to Supplementary Fig. S7. The observable changes in mitochondria signal strength is due to acquisition photobleaching corrections over time.

Supplementary Video 12
Image series to demonstrate the improved identification (segmentation) of individual mitochondria upon swelling in diH_2O (right) for organelle counting analyses, as opposed to the same cell untreated (left). The image processing steps are indicated in the top left corners. The movie corresponds to Fig. 7 b in the main article.
